# Paradoxical attenuation of early amyloid-induced cognitive impairment and synaptic plasticity in an aged APP/Tau bigenic rat model

**DOI:** 10.1186/s40478-024-01901-0

**Published:** 2024-12-20

**Authors:** Joshua T. Emmerson, Sonia Do Carmo, Agustina Lavagna, Chunwei Huang, Tak Pan Wong, Julio C. Martinez-Trujillo, A. Claudio Cuello

**Affiliations:** 1 Department of Pharmacology & Therapeutics, McGill University, McIntyre Medical Building, 3655 Promenade Sir William Osler Room 1210, Montreal, H3G 1Y6 Canada; 2 Department of Psychiatry, McGill University, Montreal, H4H 1R3 Canada; 3 Department of Physiology and Pharmacology, Schulich School of Medicine and Dentistry, Robarts Research Institute and Brain and Mind Institute, University of Western Ontario Lawson Health Research InstituteOxford University, Oxford, ON N6A 5B7 Canada; 4 Lawson Health Research Institute, Oxford, ON N6A 5B7 Canada; 5 Department of Pharmacology, Oxford University, London, ON N6A 5B7 UK

**Keywords:** Alzheimer’s disease, Amyloid beta, Animal models, Rodent behavior, Human tauopathy, Synaptic plasticity

## Abstract

The combination of amyloid beta and tau pathologies leads to tau-mediated neurodegeneration in Alzheimer’s disease. However, the relative contributions of amyloid beta and tau peptide accumulation to the manifestation of the pathological phenotype in the early stages, before the overt deposition of plaques and tangles, are still unclear. We investigated the longitudinal pathological effects of combining human-like amyloidosis and tauopathy in a novel transgenic rat model, coded McGill-R-APPxhTau. We compared the effects of individual and combined amyloidosis and tauopathy in transgenic rats by assessing the spatiotemporal progression of Alzheimer’s-like amyloid and tau pathologies using biochemical and immunohistochemical methods. Extensive behavioral testing for learning and memory was also conducted to evaluate cognitive decline. Additionally, we investigated brain inflammation, neuronal cell loss, as well as synaptic plasticity through acute brain slice electrophysiological recordings and Western blotting. Evaluation of Alzheimer’s-like amyloidosis and tauopathy, at the initial stages, unexpectedly revealed that the combination of amyloid pathology with the initial increment in phosphorylated tau exerted a paradoxical corrective effect on amyloid-induced cognitive impairments and led to a compensatory-like restoration of synaptic plasticity as revealed by electrophysiological evidence, compared to monogenic transgenic rats with amyloidosis or tauopathy. We discovered elevated CREB phosphorylation and increased expression of postsynaptic proteins as a tentative explanation for the improved hippocampal synaptic plasticity. However, this tau-induced protective effect on synaptic function was transient. As anticipated, at more advanced stages, the APPxhTau bigenic rats exhibited aggravated tau and amyloid pathologies, cognitive decline, increased neuroinflammation, and tau-driven neuronal loss compared to monogenic rat models of Alzheimer’s-like amyloid and tau pathologies. The present findings propose that the early accumulation of phosphorylated tau may have a transient protective impact on the evolving amyloid pathology-derived synaptic impairments.

**Supplementary Information:**

The online version contains supplementary material available at 10.1186/s40478-024-01901-0.

## Background

The two neuropathological hallmarks of Alzheimer’s disease (AD) are the extracellular deposition of amyloid beta (Aβ) plaques and intracellular neurofibrillary tangles (NFTs) which are composed of abnormally phosphorylated tau proteins forming paired helical filaments (PHFs). The pathological consequences of the accumulation of the Aβ peptide [79, 115] and of hyperphosphorylated tau proteins [66, 83, 92, 106] in the brain present unique aspects of disease. Interventions that individually target Aβ [29, 77, 94] or tau [39, 80, 108] have not yet yielded substantive outcomes on cognition, emphasizing the need to understand their effects in combination.

The “Trigger and Bullet” hypothesis proposes Aβ as the initiator of tau-mediated neurodegeneration [17]. While significant emphasis has been placed on the role of amyloid pathology in AD, it is ultimately the accumulation of tau that correlates more strongly with cognitive decline and brain atrophy than Aβ [4, 10, 13, 14, 42, 48, 51, 91]. Investigations in transgenic rodents demonstrated an exacerbated fibrillary tau pathology by the combination of both amyloid and tau pathologies [23, 44, 73, 104]. Preclinical studies have also demonstrated that Aβ is sufficient to provoke synaptic dysfunction in ex vivo [58, 105] and in live transgenic animal models [85, 86, 96] to elicit transmitter-specific synaptic losses (for review [11, 113]). Even at pre-plaque stages, the intraneuronal accumulation of Aβ peptides provokes cognitive deficits in APP transgenic rats [62, 71]. However, the relationship between Aβ and tau at the earliest stages of the AD pathology, such as at the synapse, remains unclear. To best investigate such an early pathological scenario, we resorted to generating a rat transgenic model displaying both the AD-like amyloidosis and tauopathy. Towards such an objective, we crossed the well-established McGill-R-Thy1-APP [52, 62, 71] and recently established McGill-R955-hTau [35, 36] transgenic (Tg) rat models, in their respective heterozygous forms, to generate a bigenic rat model with slow-progressing, human-like, amyloid and tau pathologies. Rats present several disease-relevant advantages over mice for these objectives, including endogenous expression of all isoforms of tau [50], an immune system similar to humans [22, 109], superior behavioral display [32, 67, 107] and an APOE protein with properties akin to the human APOE4 which accelerates tau-mediated neurodegeneration in vivo [95, 103].

Contrary to expectations, these studies revealed that the early combined amyloid and tau pathologies resulted in enhanced synaptic strength as evidenced by increased LTP formation compared to age-matched rats displaying only the amyloid (APP^+/−^) or the tau (R955-hTau^+/−^) pathology. Based on our subsequent investigations of these unexpected findings, we pose the possibility that a protective-like effect of the incipient tau accumulation over the initial amyloid-induced synaptic dysregulation and cognitive impairments could be mediated by a differential CREB phosphorylation and an increased generation of postsynaptic density proteins. At advanced stages, we demonstrate that the combined AD-like Aβ and tau transgenic expression resulted in increased amyloidosis and tauopathy as compared to that of the transgenic version of single pathologies, and, likewise, more marked cognitive impairments.

## Materials & methods

### Animals

Generation of the McGill-R-Thy1-APP [71] and McGill-R955-hTau [35, 36] transgenic (Tg) rat models were achieved as previously described. Tg APP rats [71] expressing human APP^751^_Swe, Ind_ under the murine Thy1.2 promoter, and, transgenic R955-hTau rats [74] expressing the longest isoform (2N4R) of human P301S mutated tau, were genetically crossed to produce the bigenic APPxhTau rats with a Wistar background. For simplification, McGill-R-Thy1-APP rats are referred to as “APP”, McGill-R955-hTau is “R955-hTau”, and the bigenic McGill-R-APPxhTau is “APPxhTau”. Only heterozygous animals were used given that heterozygous animals produce milder pathologies compared to their homozygous kin, enabling a greater sensitivity to observe early effects and to incorporate the risk factor of aging. Rat littermates were housed in groups of 3–4 in a GR1800 double-decker cage system (Techniplast, Int.) under a 12:12 light: dark cycle and were provided standard chow and water ad libitum. The use of animals for this study was approved by the McGill Animal Care Committee under the guidelines of the Canadian Council on Animal Care (CCAC).

### Experimental design

Male and female Tg rats were bred alongside wild type (Wt) littermate controls and raised in independent sets of cohorts to one of three endpoints at 12, 20 and 24 months of age (M) with a sample size of at least 10 per group per time point (Supplemental Table 1). Each end point comprised of two independently evaluated cohorts of males and females to ensure replication. At each endpoint, rats underwent behavioral testing for cognition, followed by brain perfusion and tissue collection for the subsequent examination of amyloid and tau pathologies in the brain.

### Animal behavior

Animals were habituated to handling by experimenters at least two weeks prior to behavioral testing. Assessment of cognition was achieved using a battery of behavioral tests during the beginning of the light cycle including the novel object location (NOL), novel object recognition (NOR), the social interaction (SI) test [35, 72] and Morris water maze (MWM) tasks using previously established protocols [35, 36, 49, 74]. The Y-maze task was also included to assess exploration and working memory [41]. Animal tracking data was recorded and scored with the assistance of Ethovision XT (Noldus, The Netherlands). Experimenters were blinded to genotype. To provide an overall assessment of cognitive status, as done previously [35, 36], a global cognitive index (CI) composite score was calculated by converting the behavioral score of each test component into a fraction of the maximum achievable value for each task (ranging from 0 to 1) and the values from each test were averaged across tasks for each animal.

### Y-maze

The three-armed Y shaped maze (Y-maze) task to evaluate short-term working memory and general exploratory behavior was achieved as previously described [41] with minor modification. In brief, rats were placed in a three-armed plexiglass apparatus (45 × 12 × 35 cm) diverging at 120° coated in fresh bedding mixed with soiled bedding of experimental animals, in one arm facing the end of the arm and were given 8 min to explore freely. Entries into each arm were scored when the animal placed all four limbs inside the arm. The total number of entries and number of alternations or ‘triads’ was recorded.

### Tissue collection

Rat brain tissues were collected as previously described [35]. In brief, rats were heavily anesthetized in 1% sodium pentobarbital (Equithesin) prior to transcardial brain perfusion with chilled phosphate-buffered saline. One brain hemisphere was fixed in 4% paraformaldehyde and the other hemisphere was further dissected to separate the cerebral cortex and hippocampus before being snap frozen in dry ice and stored at -80 °C until use for biochemistry.

### Immunohistochemistry and immunofluorescence

Immunohistochemical procedures on 40 μm coronal sections were done as previously described with minor modification [35]. Slices were washed in TBS and quenched in 3% hydrogen peroxide. Immediately after blocking in 10% normal goat serum (NGS) in TBS with 0.1% Triton X-100 (TBS-T), primary antibodies (Supplemental Table 3) were incubated in 5% NGS and TBS-T overnight at 4 °C on a shaker. After subsequent washes in TBS-T, slides were incubated in either mouse anti-HRP monoclonal antibody (1:30) pre-incubated with 5 µg/mL HRP (MAP/HRP kit, MediMabs, Canada) or Vectastain ABC-HRP kit (Vector Laboratories, USA) for 1 h at room temperature. Slices were washed with TBS, incubated in 6 mg/ml 3,3-Diaminobenzidine (Sigma, USA) for 10 min at room temperature, and then underwent brown chromogen activation in 1% hydrogen peroxidase. For blue chromogen, slices were further washed in TBS, then underwent incubation of goat anti-mouse and MAP-HRP, followed by visualization using the VECTOR SG substrate kit (Vector Laboratories). Stained slices were then mounted onto gelatin-coated slides and mounted using Entellan (Millipore). For immunofluorescence experiments, following blocking and primary antibody incubation as described above, slices were incubated in secondary antibody solution containing 5% NGS and TBS-T in the dark for 2 h at room temperature. Secondary antibodies were incubated at the same time. Slices were then washed and incubated in a 1ug/ul solution of 4′,6-diamidino-2-phenylindole (DAPI) for 5 min in the dark, then coverslipped using aqua polymount media (Polysciences). Co-localization and cell counting experiments was achieved by examining images containing five 1 μm Z-stacks merged into a Z-projection. Brightfield images were obtained using an Axio Imager M2 with an AxioCam506 color digital camera and ZEN v2.3 Blue imaging software (Zeiss, Germany). Fluorescence images were acquired using an LSM800 confocal laser scanning microscope (Zeiss, Germany). Image analysis of total fluorescence intensity, cell counts, and percent area was accomplished using ImageJ (NIH, USA) with consistent mask thresholds per experiment using at least two slices per animal and one to two images per region of interest per slice. Fluorescence intensity is expressed in arbitrary fluorescence units (AU).

### Quantification of amyloid beta and tau proteins by electrochemiluminescence immunoassay (ECLIA)

For Aβ, thirty milligrams of cortical brain tissue were homogenized in TBS buffer (150 mM NaCl, 50 mM Tris-HCl, 5 mM EDTA, pH 7.6) with protease inhibitors (Complete mini, Roche). Following ultracentrifugation (Beckman-Coulter Optima MAX-XP) at 100,000 g for 1 h at 4ºC, supernatants were collected and stored as the soluble fraction. Pellets were resuspended in a Tris-guanidine buffer (5 M guanidine HCl, 50mM Tris HCl, pH 8.0), spun again at 100,000 g to obtain the insoluble fraction. Supernatants were stored at -80ºC. Aβ peptides 1–42, 1–40 and 1–38 were quantified using the V-Plex Aβ peptide panel (6e10) kit (K15200E-1, Mesoscale Discovery, USA) according to manufacturer’s instructions.

Procedures for Sarkosyl-based separation of aggregating tau, as is done in human brain to isolate paired helical filaments [1] were done as previously described using 200 milligrams of cortical brain tissue [35, 36]. Total tau and total phosphorylated tau (ptau) Thr231 were quantified using the Phospho (Thr231)/Total Tau kit (#K15121D, Mesoscale Discovery, USA) and the assay was run according to manufacturer’s instructions.

### Western blot

Twenty to fifty milligrams of snap frozen hippocampal brain tissue were processed as previously described [35]. Hippocampal homogenates and Sarkosyl-based soluble and insoluble cortical fractions were analyzed by Western blot. After blocking in 5% non-fat milk or bovine serum albumin (BSA), primary antibodies (Supplemental Table 3) were incubated overnight at 4 °C with agitation. The following day, blots were incubated in a species-specific HRP-conjugated secondary antibody and bands were imaged using Western lightning plus chemiluminescence substrate (Perkin Elmer, USA) and an Amersham 600 Imager (GE Healthcare, Canada). Optical densities of protein bands of interest were obtained using ImageLab 6.0 (Bio-Rad, USA), normalized to glyceraldehyde-3-phosphate dehydrogenase (GAPDH) and expressed as fold-change of wild type littermates.

### Semi-quantitative reverse transcription polymerase chain reaction (qRT-PCR)

Total RNA content was extracted from ten milligrams of cortical and hippocampal brain homogenates using an RNEasy mini prep kit (Qiagen #74106, Germany) according to manufacturer’s instructions. RNA underwent reverse transcription into cDNA using the IScript rt-qPCR Supermix (Bio-Rad #170–8841, USA) and a standardized PCR protocol (priming at 25°C for 5 min, reverse transcription at 46°C for 20 min, inactivation at 95°C for 1 min). cDNA was stored at -20°C until use. Optimized dilutions of cDNA underwent PCR to detect the respective transgenes (hAPP: For 5’ CAG ATC CAT CAG GGA CCA AA 3’, Rev 5’ ACT GGT TGG TTG GCT TCT AC 3’; hTau: For 5’ GAA GAT GTG ACA GCA CCC TTA G 3’, Rev 5’ GTC TCC AAT GCC TGC TTC TT 3’) and was normalized against to two housekeeping genes (GAPDH: For 5’ TGA TGG GTG TGA ACC ACG AG 3’, Rev 5’ TCA TGA GCC CTT CCA CGA TG 3’; HPRT1: For 5’ CAG GCC AGA CTT TGT TGG AT 3’, Rev 5’ TCC ACT TTC GCT GAT GAC AC 3’). Relative gene expression was quantified and expressed as 2^ddCT^.

### Acute brain slice electrophysiology and induction of long-term potentiation (LTP)

Twenty-month-old rats (*n* = 4–6) were deeply anesthetized using CO_2_. After confirming unresponsiveness, rats were immediately decapitated, and forebrains were carefully extracted and mounted onto a VT1200S vibratome (Leica, Germany) with a basin containing fresh ice-cold cutting solution containing (2.5 mM KCl, 0.1 mM CaCl_2_, 4 mM MgCl, 1.25 mM KH_2_PO_4_, 26 mM NaHCO_3_, 1.6 mM glucose and 0.25 M sucrose, osmolarity of 350–360 and pH 7.35). Coronal slices 350 μm thick were then transferred into artificial cerebral spinal fluid (aCSF, 125 mM NaCl, 2.5 mM KCl, 2 mM CaCl_2_, 1 mM McCl_2_, 1.25 mM NaH_2_PO_4,_ 26 mM NaHCO_3_, 25 mM glucose, osmolarity 310–320, pH 7.35) warmed to 32 °C and allowed to stabilize for 1 h followed by further resting at room temperature for 30 min. Slices were transferred to a Faraday cabinet equipped with a slice recording chamber containing aCSF and 10 µM bicuculline methobromide to block GABAergic inputs. Slices were allowed to rest for 30 min before recording baseline evoked field excitatory post-synaptic potentials (EPSPs). A tungsten stimulating electrode was gently positioned at the CA1 *stratum radiatum* with a glass micropipette on the CA1 Schaeffer collateral downstream of the stimulating electrode. EPSPs were recorded using Clampex, Multiclamp and Clampfit software (Molecular Devices, USA). After achieving a stable baseline recording of 30 min, high frequency stimulation (HFS, 100 pulses at 100 Hz) were invoked to induce long-term potentiation (LTP). EPSPs were recorded minimally for one hour after HFS.

### Statistical analysis

Statistical analysis was performed with the assistance of GraphPad Prism 8 software (San Diego, USA) using a global alpha of 0.05. Both males and females were pooled in the analysis. Data are presented as mean +/- standard error of the mean (SEM). Each time point and each brain region were analyzed independently. Two-tailed unpaired t-tests were performed on single factor, single comparisons. One-way and Two-way ANOVA, as appropriate, were followed up with Bonferroni’s adjustment for three or less comparisons, and Holm-Sidak’s multiple comparisons otherwise. One-way and two-way repeated measures ANOVA (RM-ANOVA) were followed up with Holm-Sidak’s pairwise comparisons using a mixed design. If a significant interaction was found, only simple main effects of genotype were examined. Statistical outliers were removed from datasets using Grubbs test.

## Results

### Tau accelerates amyloid pathology during advanced stages of plaque deposition

**Fig. 1:**
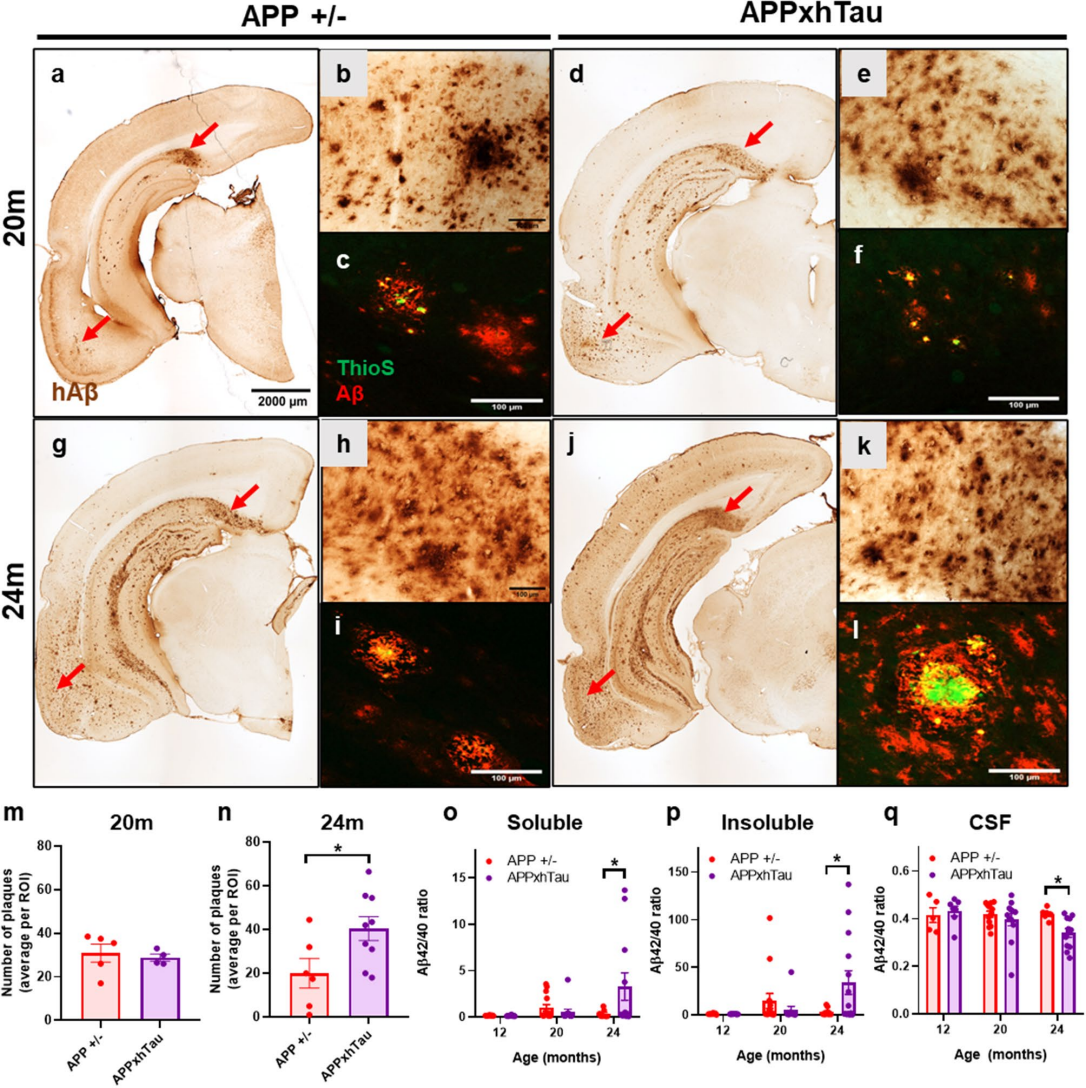
Amyloid plaque pathology in heterozygous APP and APPxhTau transgenic rats. **(a**, **d**, **g**, **j)** Representative micrographs of coronal brain sections illustrating human amyloid beta (Aβ) immunoreactivity in the caudal hippocampus and cerebral cortex at 20 (**a**-**f**) and 24 months of age (**g**, **l**). Note that plaque deposition initially appears in the subiculum and entorhinal area (red arrows). (**b**, **e**, **h**, **k**) Representative micrographs of the subiculum region of the hippocampus illustrating the appearance of diffuse and dense core plaques, and higher abundance of dense core plaques in 24-month-old bigenic rats. (**c**, **f**, **i**, **l**) Representative immunofluorescence micrographs illustrating aggregated amyloid material as shown by the colocalization between ThioflavinS (This) and Aβ in the subiculum. Quantification of the number of plaques in the subiculum at 20 **(m)** and 24 months of age **(n)** per region of interest (ROI). (**o-q**) Quantification of the Aβ42/40 ratio in the soluble (**o**) and insoluble brain fractions (**p**) as well as in cerebrospinal fluid (CSF) (**q**) showing an acceleration of Aβ peptides in 24-month-old APPxhTau rats as compared to APP^+/−^ single transgenics (*n* = 5–9). Scale bar **a**, **d**, **g**, **j** = 2000 μm; **b-c**, **e-f**, **h-i**,** k-l** = 100 μm. * *p* < 0.05; ** *p* < 0.01

The progressive human amyloid pathology in APP^+/−^ transgenic (Tg) rats was examined and compared to APPxhTau rats at 12, 20 and 24 months of age (M). At 12 M, both APP^+/−^ and APPxhTau Tg rats exhibited similar magnitudes of human intracellular Aβ immunoreactivity (IR) in the hippocampus and cerebral cortex as revealed by the human Aβ specific monoclonal antibody McSA1 [47] (Fig. S1b-c). By 20 M, approximately one third of the APP^+/−^ and APPxhTau rats developed diffuse plaques (Supplemental Table 2) in the entorhinal area and more in the subiculum region of the hippocampus (Fig. 1a-b, d-e). However, no differences in the number of plaques between APP^+/−^ and APPxhTau rats was observed in the subiculum (Fig. 1m), nor in other regions of the hippocampus (Fig. S1e) and cerebral cortex (Fig. S1f). Scarcely, plaques in the subiculum showed dense core properties as shown by ThioflavinS-immunoreactive particles colocalized with Aβ (Fig. 1c, f).

At 24 M, APP^+/−^ and APPxhTau rats developed more advanced plaque pathology as shown by an overall increased abundance and spread of plaques into other cortical and caudal hippocampal regions (Fig. 1g, j). Further, half of the APPxhTau rats developed cortical plaques compared one third in APP^+/−^ rats, suggestive of elevated cortical amyloid pathology (Supplemental Table 2). There were no differences in the number of rats with hippocampal plaque deposition. In the subiculum, APPxhTau rats developed approximately two-fold more plaques than APP^+/−^ rats (Fig. 1n). Further, increased density of ThioflavinS immunofluorescent material was observed, indicating elevated density of mature plaques (Fig. 1l). Within other brain regions, there were no differences in the total number of plaques within the hippocampus (Fig. S1g) and cerebral cortex (Fig. S1h).

Quantification of Aβ peptides at 12, 20 and 24 M revealed a marked increase in the Aβ42/40 ratio in APPxhTau rats as compared to APP^+/−^ at 24 M for both the soluble (Fig. 1o) and insoluble fractions (Fig. 1p) but not at earlier time-points. Further, a decrease in the Aβ42/40 ratio in the CSF, as is observed in human AD [19, 40, 99], was detected in APPxhTau compared to APP^+/−^ rats (Fig. 1q), and overall paralleled the increased CNS plaque pathology. Investigation of the transcript and protein levels of APP as well as the protein levels of the soluble APP alpha (sAPPα) fragment showed that total APP (22C11) protein levels were elevated by approximately 3-4-fold in both APP^+/−^ and APPxhTau rats compared to wild type (Wt) rats at 20 M (Fig. S2a-b) and 24 M (Fig. S2a, c). Further, APP protein expression in APPxhTau was similar to that of APP^+/−^ Tg rats at both time points (Fig. S2b-c). To rule out gene expression changes as a cause for pathology, we evaluated the gene expression of the human APP transgene by qRT-PCR, which revealed no differences between APP^+/−^ and APPxhTau rats (Fig. S2g). Consistently, levels of the neuroprotective sAPPα protein fragment were not significantly different between APP^+/−^ and APPxhTau rats at 20 (Fig. S2e) and 24 M (Fig. S2f). Altogether, the above evidence demonstrates that combining mild expression of human tau with amyloid exacerbated the advanced, but not initial, amyloid pathology in this rat model.

### Amyloid facilitates downstream tau hyperphosphorylation and oligomerization

**Fig. 2:**
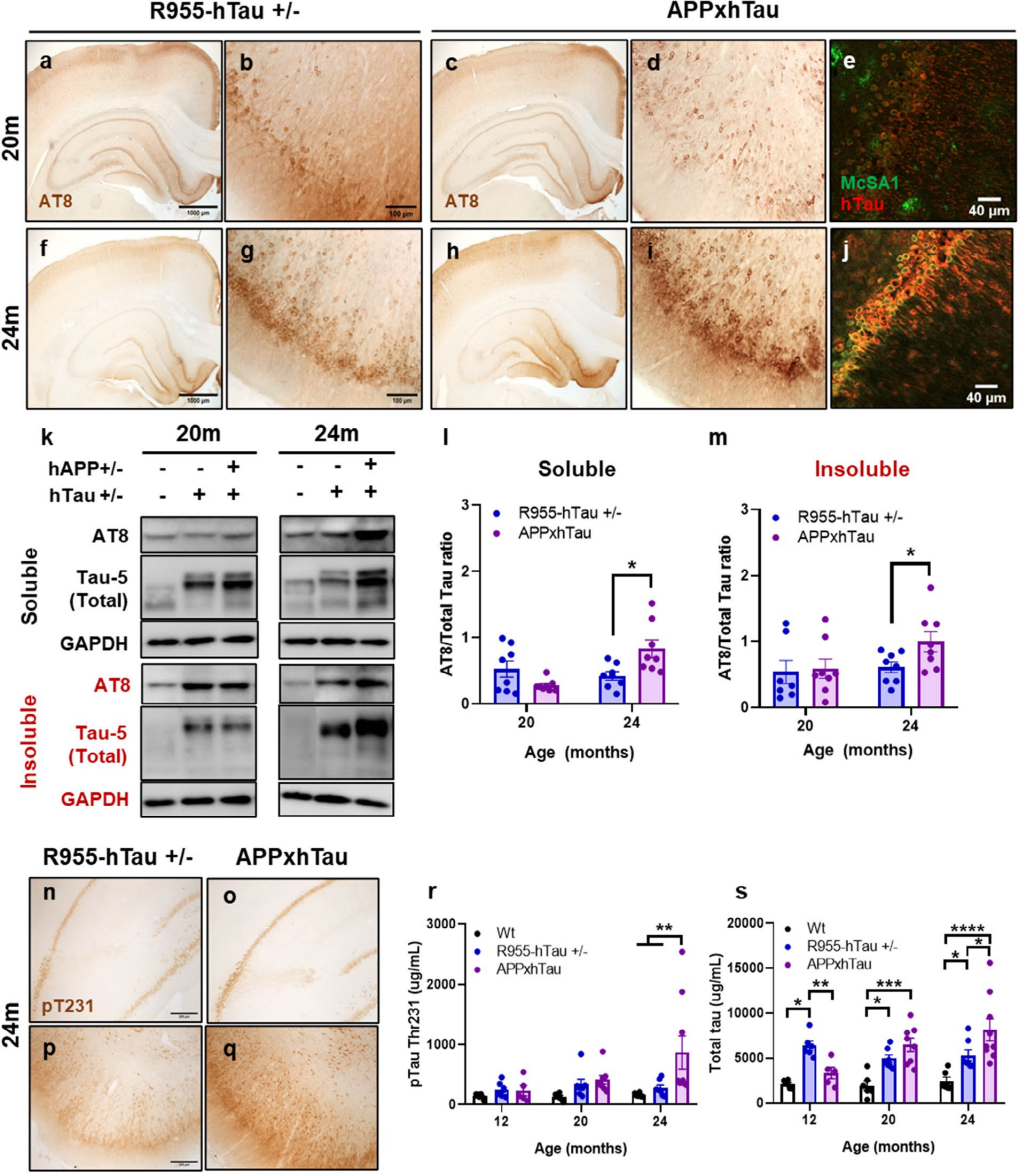
Acceleration of tau hyperphosphorylation at pSer202-Thr205 and pThr231 in 24-month-old APPxhTau rats. (**a**, **c**, **f**, **h**) Representative micrographs illustrating pSer202-Thr205 immunoreactivity in the cerebral cortex and hippocampus as probed using the monoclonal antibody AT8 and in the entorhinal area (**b**, **d**, **g**, **i**), in neurons at 20 (**a-d**) and 24 months of age (**f-i**). In APPxhTau rats, colocalization of human amyloid beta and total human tau was detected in the most severe cases as shown by representative confocal micrographs at 20 (**e**) and 24 months of age (**j**). (**k**) Representative Western blots of cortical brain homogenates from the Sarkosyl-soluble (**l**) and Sarkosyl-insoluble (**m**, labelled in red) fractions in 20 and 24-month-old rats. Quantification of ptau at Ser202-Thr205 revealed increased Sarkosyl-insoluble tau protein in APPxhTau rats at 20 months as well as at 24 months in the Sarkosyl-soluble and insoluble tau. **(n-q)** Representative micrographs of the CA1-CA2 region of the hippocampus (**n-o**) and the entorhinal cortex (**p-q**) at 24 months of age illustrating elevated ptau Thr231 immunoreactivity in APPxhTau rats as compared to R955-hTau^+/−^ single transgenics. Quantification of Sarkosyl-insoluble ptau Thr231 (**r**) and total tau (**s**) by electrochemiluminescence assays (ECLIA) revealed increased accumulation of tau proteins in 24-month-old APPxhTau rats as compared to wild type and R955-hTau^+/−^ rats (*n* = 6–9). Scale bar **a**,** c**,** f**,** h** 1000 μm; **b**,** d**,** g**,** i** 100 μm **q-t** 200 μm. * *p* < 0.05; ** *p* < 0.01; ***p < 0.001; ****p < 0.0001

The initial human-like tauopathy in R955-hTau^+/−^ rats was previously evidenced by a subtle increase in ptau Ser202-Thr205 (AT8 monoclonal antibody (mAb)) IR after 18 M followed by a surge of ptau Ser202-Thr205, pThr231 and oligomeric tau after 24M [36]. By comparison, 20-month-old R955-hTau^+/−^ and APPxhTau rats exhibited a similar distribution of AT8 IR in neurons of the cerebral cortex and hippocampus (Fig. 2a, c), including the entorhinal cortex (Fig. 2b, d) and subiculum (Fig. S3a-b). Interestingly, confocal analysis of samples dual immunolabelled for human Aβ (McSA1 IR) and tau (AT8 IR) revealed a colocalization of Aβ and ptau in 24-month-old APPxhTau rats. This colocalization was solely present in rats with severe pathology and was not observed in rats with milder pathology or at 20 M (Fig. 2e, j). Protein levels of ptau Ser202-Thr205, as examined by Western blot after Sarkosyl-based separation, were similar across genotypes in the Sarkosyl-soluble fraction (Fig. 2k-l). In contrast, Sarkosyl-insoluble pSer202-Thr205 IR, normalized to total tau protein detect by Tau-5, was elevated significantly in APPxhTau, but not R955-hTau^+/−^ rats, as compared to Wt rats (Fig. 2k, m), suggesting a ramping up of tau hyperphosphorylation and oligomerization attributed to amyloid plaque pathology. When AT8-IR was normalized to total protein by GAPDH and to wild type rats to account for nonspecific reactivity, the results trended the same (Fig. S3e-f). During more advanced plaque pathology at 24 M, ptau Ser202- Thr205 was elevated in APPxhTau rats compared to R955-hTau^+/−^ rats as shown by an increased burden of AT8 IR in neurons of the cortex and hippocampus (Fig. 2g, i). Accordingly, AT8 IR was also increased in cortical homogenates of APPxhTau rats in both Sarkosyl-soluble (Fig. 2l) and Sarkosyl-insoluble fractions (Fig. 2m), demonstrating accelerated ptau for an epitope known to become compromised in early stages in AD.

Immunoreactivity for ptau Thr231 (AT180 mAb), was only detected at 24 M and showed elevated IR in the CA1-CA2 region of the hippocampus (Fig. 2n-o) and the entorhinal cortex (Fig. 2p-q) of APPxhTau rats as compared to R955-hTau^+/−^ rats. Accordingly, ultrasensitive electrochemiluminescence assays (ECLIA) revealed that ptau Thr231 was 2-3-fold higher in the Sarkosyl-insoluble fraction of APPxhTau rats as compared to R955-hTau^+/−^ rats at 24 M, whereas no differences were found at younger ages (Fig. 2r). Examination of total tau levels in Sarkosyl- soluble and insoluble fractions by ECLIA revealed early increases of Sarkosyl-insoluble total tau in R955-hTau^+/−^, but not APPxhTau as compared to Wt rats at 12 M (Fig. 2s). By 20 M, Sarkosyl- insoluble total tau protein was similarly elevated in both R955-hTau^+/−^ and APPxhTau rats. Interestingly, at 24 M, Sarkosyl-insoluble total tau was 1.5-fold higher in APPxhTau rats as compared to R955-hTau^+/−^ rats (Fig. 2s). In contrast, in Sarkosyl-soluble fractions, both ptau Thr231 (Fig. S3g) and total tau (Fig. S3h) peaked at approximately 2-3-fold higher than Wt rats with no detectable differences between R955-hTau^+/−^ and APPxhTau rats, regardless of age. The differences in tau levels were not due to differences in tau expression since tau gene expression, as assessed by qRT-PCR, was not significantly different between R955-hTau^+/−^ and APPxhTau rats at any age (Fig. S3i). Furthermore, tau pathology in APPxhTau rats did not produce overt NFTs as demonstrated by the absence of misfolded pre-tangle tau (MC1 mAb) or overt pSer396-Ser404 (PHF1 pAb) c-terminal ptau immunoreactivity (data not shown). Altogether, this signifies a progressive acceleration of tau pathology driven by amyloid pathology.

### Aβ-induced impairments in cognition are initially counteracted by tau accumulation

**Fig. 3:**
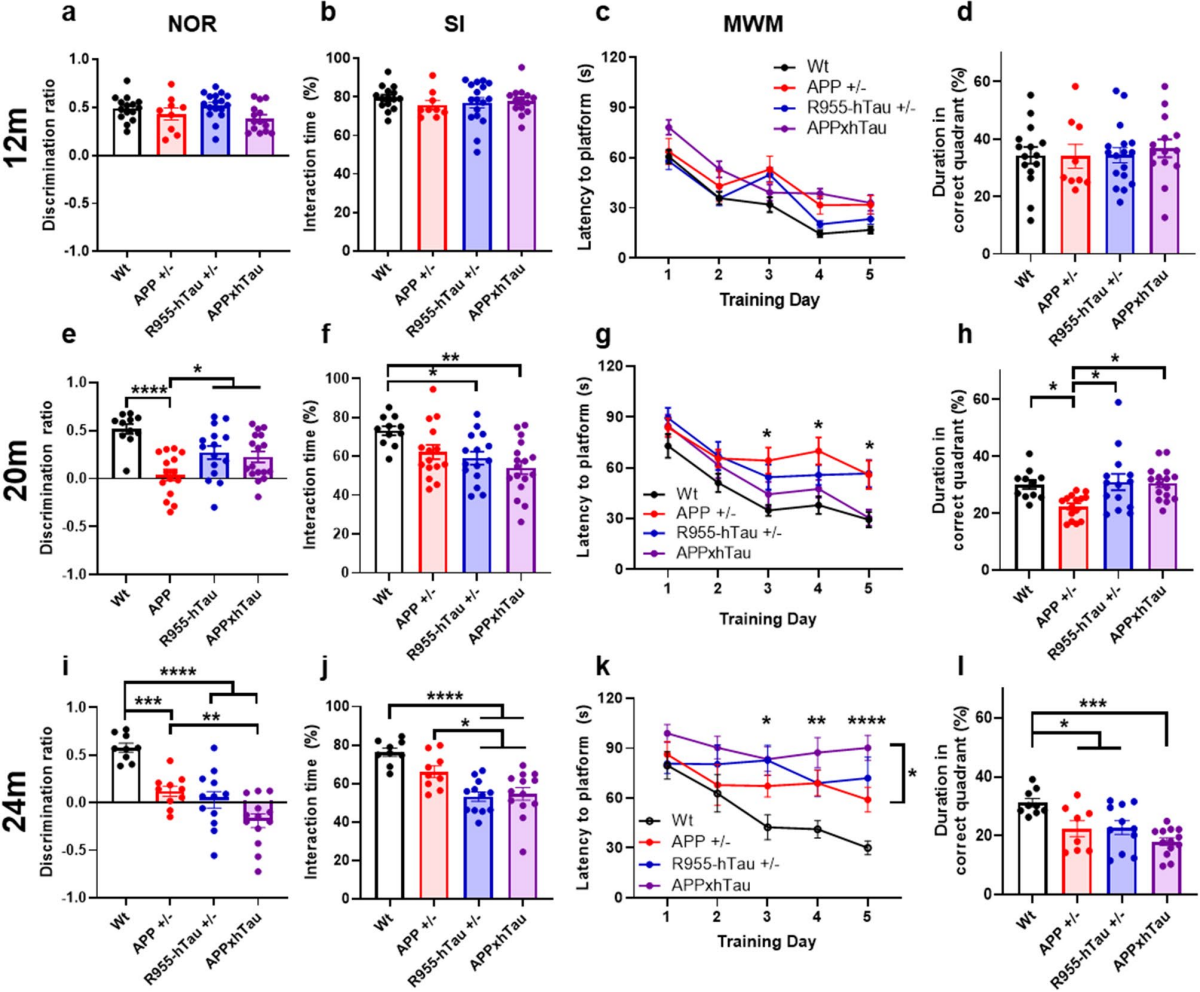
Tau dictates cognitive status in APPxhTau rats with an initial compensatory effect followed by exacerbation of impairments. Evaluation of cognition using a panel of behavioral tests conducted in cohorts of rats at 12 (**a**-**d**), 20 (**e**-**h**) and 24 months of age (**i**-**l**) including discrimination of a novel object during the novel object recognition (NOR) (**a**, **e**, **i**), interaction time with an age- and sex-matched unfamiliar conspecific during the three-chamber social interaction task (**b**, **f**, **j**), and time required to find the hidden platform during the training phase of the Morris water maze (**c**, **g**, **k**) and the probe trial (**d, h, l**). At 20 months of age, the amyloid-derived impairments in APPxhTau rats during the NOR (**e**) and MWM tasks (**g**) are compensated by tau. Note that performance of APPxhTau rats during the MWM (**g**) is similar to that of Wt rats whereas single transgenic APP^+/−^ and R955-hTau^+/−^ rats show impairments on days 3, 4 and 5. At 24 months, the NOR (**i**) and MWM (**k**) tasks show significantly lower scores in APPxhTau rats as compared to APP^+/−^ rats (*n* = 8–15). * *p* < 0.05, ** *p* < 0.01, *** *p* < 0.001, **** *p* < 0.0001

Given that the combined presence of amyloid and tau facilitated the development of their respective pathological phenotype, we then examined their merged effect on cognition. Unexpectedly, our findings revealed that the combination of amyloid and tau acts as a double-edged sword on cognitive abilities. At first, their combination is seemingly protective against Aβ-induced cognitive decline to then accelerate it at late pathological stages. In more detail, there was a clear and early effect attributed to amyloid on cognition in APP^+/−^ rats. At 12 M, minor cognitive impairments could be detected in APP^+/−^ rats but not in R995-hTau^+/−^ and APPxhTau rats. As such, 12-month-old APP^+/−^ rats, which display iAβ and no amyloid plaque deposition, presented an impaired ability to discriminate the relocated object during the novel object location (NOL) task (Fig. S4a). However, at this time-point none of the Tg rat groups showed impairments during the other investigated behavioral tasks compared to Wt rats (Fig. 3a-d, S4a-b), including the discrimination ratio (DR) during the novel object recognition (NOR) (Fig. 3a), interaction time during the social interaction task (SI) (Fig. 3b), latency to find the hidden platform during the training (Fig. 3c) and probe trials of the Morris water maze (MWM) (Fig. 3D) as well as the percentage of alternations during the Y-maze (Fig. S4b). Compiling the scores from all behavioral tasks into a global cognitive index (CI) composite score translated into a 20% reduction in overall cognitive performance in 12-month-old APP^+/−^ compared to Wt rats (Fig. S4c) while APPxhTau rats showed a cognitive index like that of Wt and R955-hTau^+/−^ rats.

At 20 M, the negative effects of Aβ on cognition worsened. APP^+/−^ rats were impaired in all tasks employed except for the SI task where statistical significance was not reached. At this time-point, cognitive impairments also became apparent in R955-hTau^+/−^ rats. R955-hTau^+/−^ rats presented a reduced DR during the NOL task (Fig. S4d), a reduced interaction time in the SI task (Fig. 3f) as well as an increased latency to platform in the acquisition phase of the MWM (Fig. 3g). However, they performed significantly better than APP^+/−^ rats in other tasks including the NOR (Fig. 3e), Y-maze (Fig. S4e), and the probe test of the MWM (Fig. 3h). Consequently, the CI was severely reduced in APP ^+/−^ compared to Wt rats but was elevated in comparison to R955-hTau^+/−^ rats (Fig. S4f).

Unexpectedly, APPxhTau rats performed similarly to R955-hTau^+/−^ rats but distinctly better than APP^+/−^ rats in the NOR task (Fig. 3e). This apparent compensation of behavioral performance by tau was also observed during the Y-maze as shown by a partially abolished percentage of alternations in APPxhTau rats (Fig. S4e). The SI task revealed social withdrawal behaviors in Tg rats harbouring the tau transgene, shown by a significant reduction in the interaction time in R955-hTau^+/−^ and APPxhTau rats (Fig. 3f). Most strikingly, APPxhTau rats performed similarly to the cognitively unimpaired Wt rats during the acquisition phase and the probe test of the MWM, markedly contrasting in the impaired APP^+/−^ rats (Fig. 3g-h). However, the combination of the behavioral outcomes in the CI composite score obscured these differences indicating an overall reduced performance in all Tg animals by 40% compared to Wt.rats (Fig. S4f). In APPxhTau rats, the negative effects from Aβ on cognitive performance were counteracted by tau at stages resembling the initial Aβ plaque deposition. As expected, at the latest time point of 24 M, multiple and more advanced deficits in cognition were detected in all Tg rat groups compared to Wt rats. Further, APPxhTau rats presented significantly worse cognitive measures compared to APP^+/−^ rats in multiple tasks including in the NOL (Fig. S4g), NOR (Fig. 3i), SI (Fig. 3j), and the acquisition phase of the MWM (Fig. 3k), with a trended decrease in the time spent in the correct quadrant during the probe test (Fig. 3l). This was reflected by a lower CI indicating that cognitive status in APPxhTau rats was 65% lower compared to Wt rats, whereas APP^+/−^ and R955-hTau^+/−^ rats were 40% lower compared to Wt rats (Fig. S4i). This confirms an exacerbation of cognitive impairments when both pathologies are present and simultaneously worse.

### Tau accumulation transiently counteracts Aβ-induced impairments in synaptic plasticity

**Fig. 4:**
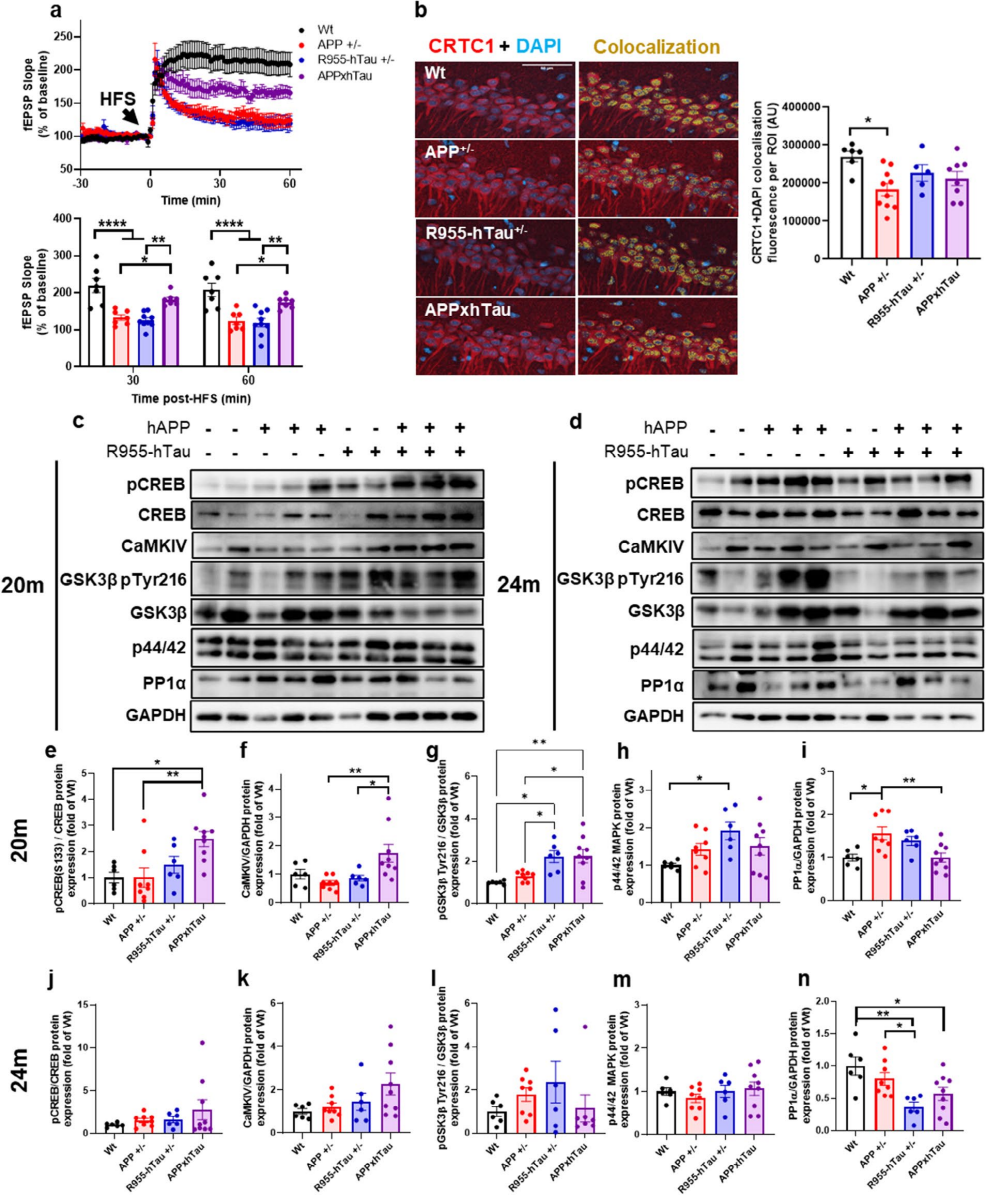
Tau counteracts amyloid-induced deficits in synaptic plasticity and induces distinct alterations to CREB phosphorylation in 20-month-old APPxhTau rats. Acute brain slice recordings of field excitatory postsynaptic potentials (fEPSP) slope expressed as a percentage of baseline (**a**). Quantification of fEPSP 30–60 min after HFS demonstrating impaired responses after high frequency stimulation (HFS) in 20-month-old APP^+/−^ and R955-hTau^+/−^ rats and a partially abolished response in APPxhTau rats. (**b**) Quantification of CRTC1 and DAPI colocalization fluorescence intensity in the CA1 region of the hippocampus. (**c**-**d**) Representative Western blots of hippocampal homogenates illustrating alterations to CREB phosphorylation at Ser 133 as well as protein expression of established protein kinases and phosphatases of CREB at 20 (**c**,** e**-**i**) and 24 months of age (**d**, **j**-**n**). Quantification of Western blots for pCREB (e, j), CaMKIV (f, k), pGSK3β Tyr216 (g, l), p44/42 MAPK (Erk1/2) (**h**, **m**), and PP1α (**i**, **n**) illustrating early elevations in protein expression of kinases such CaMKIV and GSK3β as well as phosphatases such as PP1α in 20-month-old APPxhTau rats (*n* = 6–9). * *p* < 0.05, ** *p* < 0.01, **** *p* < 0.0001

To confirm and further investigate the unexpected amelioration in cognitive performance observed in 20-month-old APPxhTau rats, we performed acute brain slice electrophysiology to induce long-term potentiation (LTP) in the CA1 region of the hippocampus. Twenty-month-old APP^+/−^ and R955-hTau^+/−^ rats exhibited an impaired LTP response after high frequency stimulation (HFS) compared to Wt rats, as shown by a decaying of field excitatory post-synaptic potential (fEPSPs) that returned to baseline as early as 30 min post-HFS (Fig. 4a). Interestingly, the combination of APP^+/−^ and R955-hTau^+/−^ transgenes produced a partial rescue in synaptic function as shown by restoration of fEPSP strength in APPxhTau, significantly elevated compared to APP^+/−^ and R955-hTau^+/−^ rats.

We then examined cellular pathways potentially explaining the rescue of Aβ-induced impairments by tau. The phosphorylation of Cyclic response element binding protein (pCREB) at Ser133 is critical for dynamically mediating synaptic plasticity in the hippocampus [16, 30, 88] and results in translocation of CREB-regulated transcription in response to neuronal activation. Nuclear CRTC1 immunofluorescence was defined as the signal which overlapped with DAPI staining for heterochromatin. We found that CA1 hippocampal neurons of APP^+/−^ rats showed a decrease in nuclear CRTC1 compared to Wt rats as shown by a reduction in CRTC1 colocalization with DAPI (Fig. 4b). Such a reduction was not found in R955-hTau^+/−^ rats. In addition, levels of nuclear CRTC1 in APPxhTau rats were similar to Wt and R955-hTau^+/−^ rats, suggesting a tau-mediated rescue of Aβ-induced impairments in synaptic plasticity and potential stabilization of pCREB/CBP/CRTC1 complexes to engage gene transcription. Furthermore, Western blotting on hippocampal homogenates revealed that at 20 M, the pCREB Ser133/Total CREB ratio was significantly elevated in APPxhTau and R955-hTau^+/−^ rats but not in APP^+/−^ rats as compared to Wt littermates (Fig. 4e) whereas total CREB expression was unaffected (Fig. S5e). Further investigation of protein kinases and phosphatases that regulate pCREB Ser133 was then performed. Interestingly, Calcium-calmodulin kinase IV (CaMKIV), the most important Ca2+-activated CREB kinase in vivo which is activated by sustained synaptic excitation, was elevated only in APPxhTau rats (Fig. 4f) whereas Calcium-calmodulin kinase II alpha (CaMKIIα) protein expression was unchanged (Fig. S5f). Glycogen synthase kinase 3 beta (GSK3β), also known as a prominent tau kinase and inhibitor of CREB activity, exhibited elevated phosphorylation at Tyr216 in 20-month-old APPxhTau and R955-hTau^+/−^ rats as compared to APP^+/−^ and wild type rats (Fig. 4g). pGSK3β at Ser9 trended similarly with a possible synergy in APPxhTau rats (Fig. S5g). In contrast, p44/42 mitogen-activated protein kinase (p44/42MAPK, Erk1/2) that signals to an independent set of CREB kinases (RSK1-3 and MSK1/2) was elevated only in R955-hTau^+/−^ rats (Fig. 4h). Phosphatases of CREB such as Protein phosphatase 1 alpha (PP1α) was elevated in APP^+/−^ but was abolished to baseline in APPxhTau rats (Fig. 4i) whereas calcineurin (PP2B) was unaltered (Fig. S5h). However, p38MAPK, another tau kinase affiliated with synaptic plasticity that signals to CREB through MSK1, was unaltered in 20 M transgenic rats (Fig. S3k). Further to it, our findings suggest that the observed beneficial interaction between amyloid and tau in stimulating CREB/CRTC1 signaling at 20 M is mediated by a fine balance between CaMKIV and PP1α effects with a contribution of GSK3β. At 24 M, such differences in the pCREB ratio (Fig. 4j), total CREB (Fig. S5k) and CaMKIV protein expression were absent (Fig. 4k). However, p38MAPK were significantly increased by 2-3-fold in APPxhTau as compared to APP^+/−^ and Wt rats (Fig. S3l). Similar to the observations at 20 M, CaMKIIα showed no differences across groups (Fig. S5l), Interestingly, PP1α was significantly reduced in R955-hTau^+/−^ and APPxhTau rats as compared to Wt (Fig. 4n), whereas pGSK3β (Fig. S5m) and PP2B (Fig. S5n) was unaffected in 24-month-old rats. This emphasizes that the observed changes pertaining to CREB phosphorylation at 20 M were transient.

**Fig. 5:**
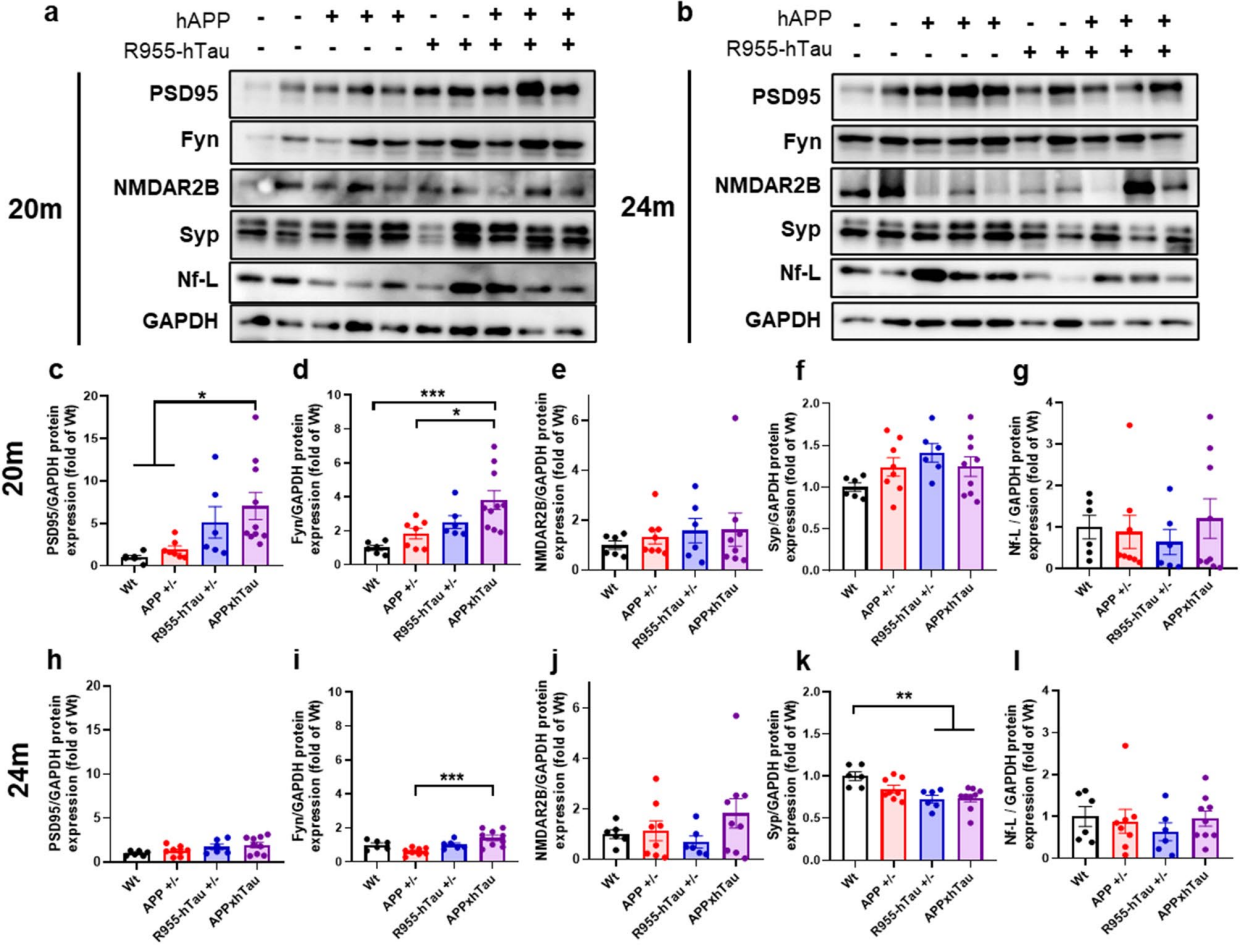
Combination of amyloid and tau alters postsynaptic density proteins involved with NMDA receptor activity (**a-b**) Representative Western blots on hippocampal homogenates of 20-month-old (**c**) and 24-month-old rats (**d**) illustrating elevated postsynaptic density 95 (PSD-95) (**c**) and Fyn (**d**) protein expression in 20-month-old APPxhTau rats as compared to APP^+/−^ and wild type rats, whereas NMDAR2B (**e**), Synaptophysin (**f**, Syp) and neurofilament light chain (**g**, Nf-L) expression are unaffected. At 24 months, PSD-95 (**h**) and Fyn (**i**) are elevated in APPxhTau rats as compared to wild type and APP^+/−^ rats. NMDAR2B (**j**) remained at levels similar to wild type rats. Syp protein expression was diminished in R955-hTau^+/−^ and APPxhTau rats (**k**) whereas Nf-L expression was unaffected (**l**) (*n* = 6–9). * *p* < 0.05, ** *p* < 0.01, ***p < 0.001

Next, we investigated postsynaptic proteins involved in the LTP response including the N-methyl-D-aspartate receptor (NMDAR) as well as the post-synaptic density protein (PSD-95) and the Src kinase Fyn, which are proteins involved in modulating NMDARs, using Western blot on hippocampal homogenates (Fig. 5a-b). At 20 M, the protein levels of PSD-95 (Fig. 5c) and Fyn (Fig. 5d) were increased by 6- and 4-fold, respectively, in APPxhTau compared to Wt and APP^+/−^ rats, while no differences in overall NMDAR expression were detected for the NMDAR2B (Fig. 5e), NMDAR1 (Fig. S5c), nor NMDAR2A subunits (Fig. S5d). Investigation of presynaptic markers showed no significant changes at 20 M, including synaptophysin (Syp) as a marker of presynaptic density (Fig. 5f) and neurofilament light chain (Nf-L) for axonal degeneration (Fig. 5g), indicating no overt changes in presynaptic vesicles or overt neurodegeneration. During advanced plaque pathology and hyperphosphorylated tau at 24 M, PSD-95 levels were not significantly different (Fig. 5h). In contrast, Fyn levels were marginally elevated in APPxhTau rats compared to APP^+/−^ rats, with a lesser magnitude than that observed at 20 M (Fig. 5i). As expected, a tau-mediated decrease in synaptophysin was observed in both R955-hTau^+/−^ and APPxhTau rats (Fig. 5k). However, no changes in Nf-L were detected (Fig. 5l) nor NMDAR2B (Fig. 5j), NMDAR1 (Fig. S5i) and NMDAR2A (Fig. S5j), resembling a human-like presynaptic density loss preceding neurodegeneration. Altogether, our findings suggest that in addition to its effects on CREB/CRTC1 signaling, the rescue of Aβ-induced deficits by tau at 20 M is mediated in part by PSD-95 and Fyn signaling.

**Fig. 6:**
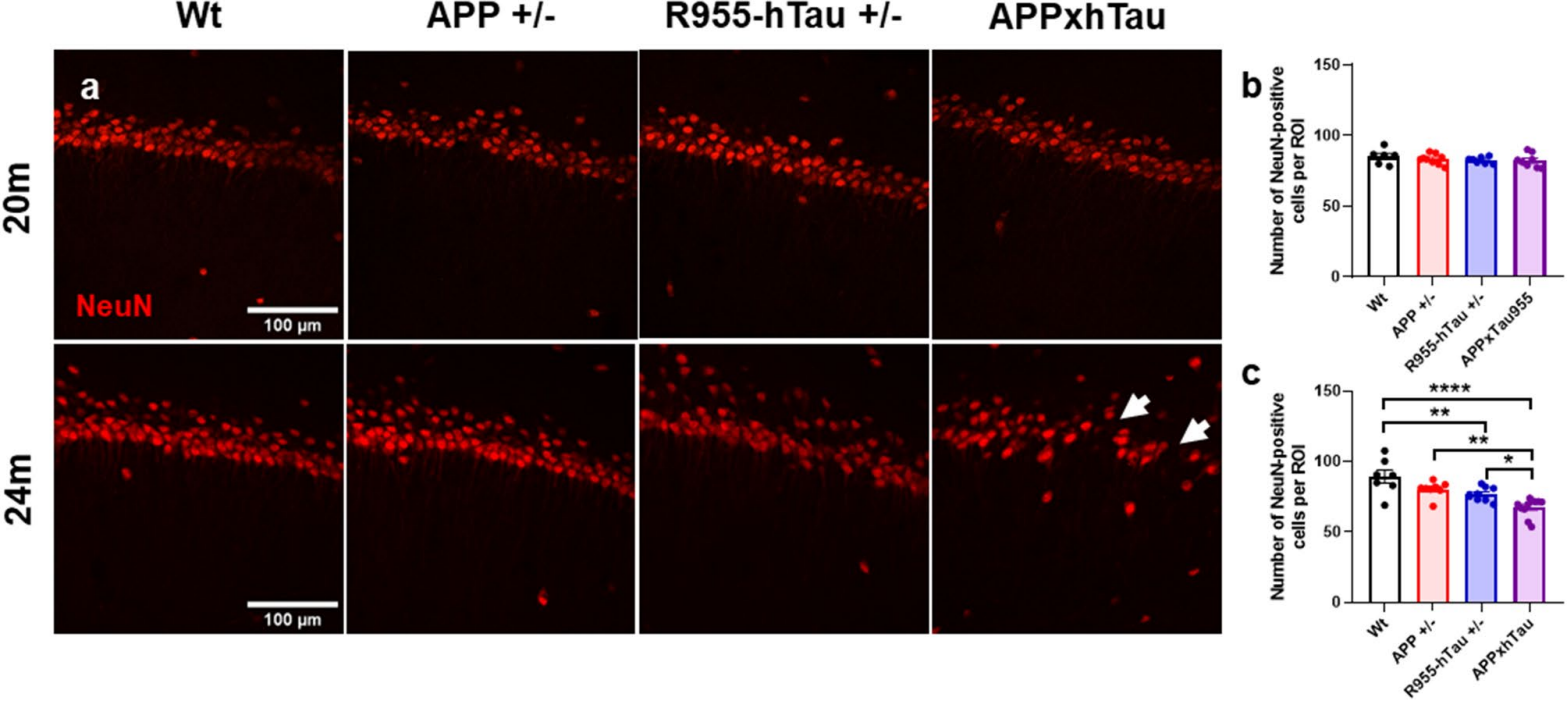
Accelerated neuronal loss and hippocampal disorganization in 24-month-old APPxhTau rats. (**a**) Representative immunofluorescence micrographs of the CA1 region of the hippocampus at 20 and 24 months of age illustrating the disrupted organization of neurons in R955-hTau^+/−^ and APPxhTau rats at 24 months (white arrows). Quantification of the total number of NeuN-immunolabelled neurons per region of interest (ROI) in the CA1 at 20 (**b**) and 24 (**c**) months of age demonstrating a significant reduction of neurons in R955-hTau^+/−^ rats, with greater losses in APPxhTau rats (*n* = 6–9). Scale bar: **a** 100 μm

### Enhanced tau-driven hippocampal neuronal loss in aged APPxhTau rats

R955-hTau^+/−^ rats develop neuronal loss in the CA1 after 24M [36] while neuronal loss is absent in APP^+/−^ rats. To determine whether tau-driven degeneration is accelerated in APPxhTau rats we examined the number of NeuN-IR cells in the CA1 and subiculum regions of the hippocampus and in the entorhinal cortex. At 20 M (Fig. 6a), there was no neuronal loss in any of the brain regions examined in any of the rat groups (Fig. 6b, S6a-b). At 24 M, our results showed a marked reduction in the number of NeuN-immunofluorescent labelled neurons in the CA1 of APPxhTau rats as compared to Wt (~ 25%), APP^+/−^ (~ 25%) and to R955-hTau^+/−^ (~ 15%) rats (Fig. 6c). This neuronal loss was accompanied by a disorganization of the neuronal layers. As expected, neuronal loss was also present in the CA1 of R955-hTau^+/−^, but not in APP^+/−^ rats (Fig. 6c). A significant neuronal loss was also evident in the entorhinal cortex of APPxhTau rats (Fig. S6d), indicating an amplification of tau-driven degeneration by amyloid.

**Fig. 7:**
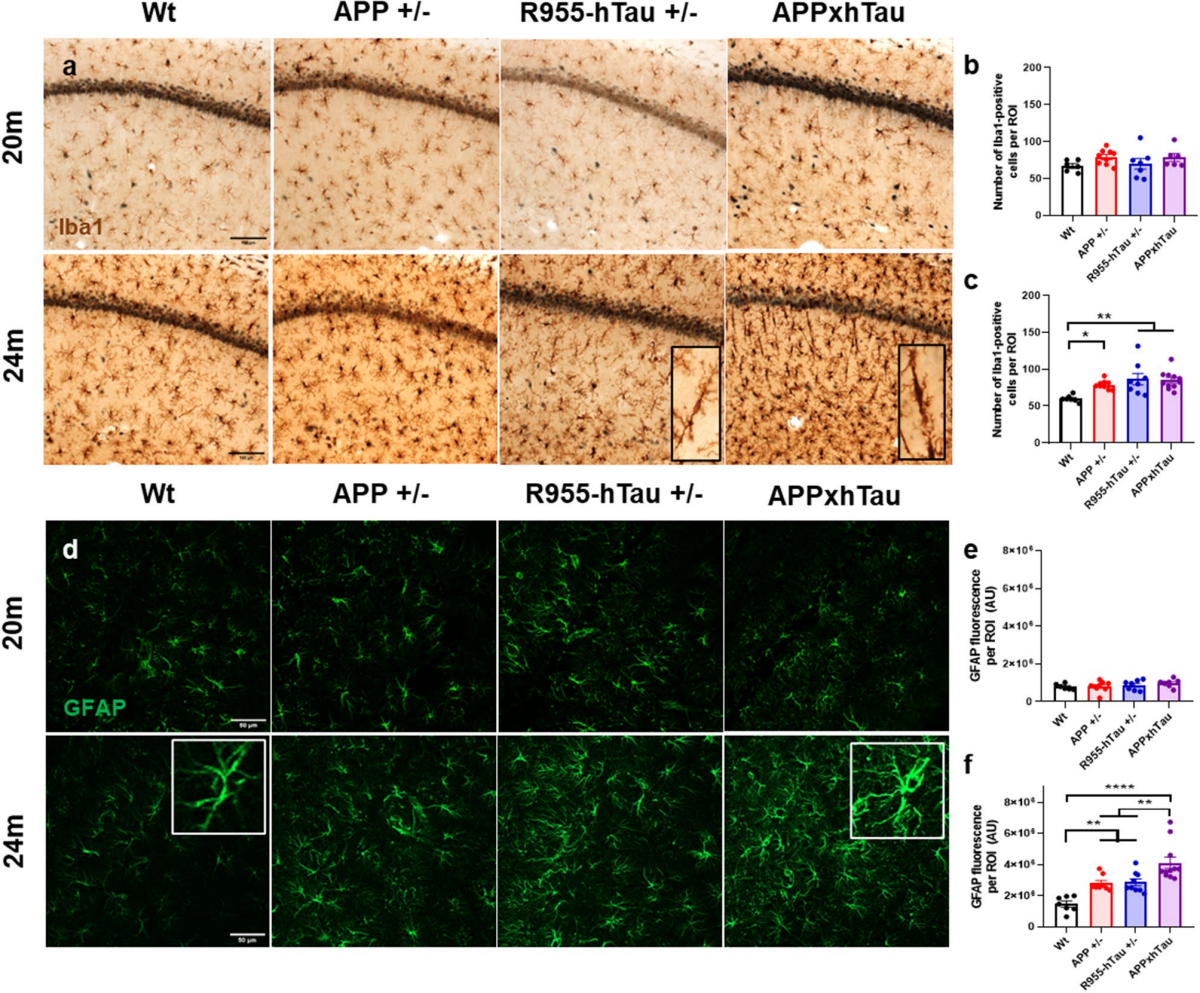
Transgenic APP^+/−^ and R955-hTau^+/−^ rats exhibit robust inflammatory responses in microglia and astrocytes with downstream additive effects on astrocyte reactivity. (**a**) Representative micrographs of microglial cell abundance in the CA1 region of the hippocampus, as shown by Iba1 immunoreactivity (brown) in APP^+/−^, R955-hTau^+/−^ and APPxhTau transgenic rats as compared to wild type (Wt) rats at 20 and 24 months of age. At 24 months of age, a CNS inflammatory response and microgliosis was detected as quantified by an increase in the total number of Iba1-immunorective cells (**c**) but not at 20 months (**b**) including the occurrence of rod-shaped microglia. (**d**) Representative immunofluorescence micrographs of the glial fibrillary acidic protein (GFAP) at 20 and 24 months of age illustrating an elevation and exacerbated astrocytic fluorescence intensity in the CA1 of 24-month-old APPxhTau rats as compared to wild type and single APP^+/−^ and R955-hTau^+/−^ transgenic rats (*n* = 6–10). Scale bar: **a** 100 μm; **d** 50 μm. * *p* < 0.05, ** *p* < 0.01, **** *p* < 0.0001

### Synergistic effects of amyloid and tau on microglia and astrocyte reactivity

As a CNS inflammatory response plays a principal component in amyloid and tau pathologies, we examined whether their combination would amplify glial activation in APPxhTau rats using quantitative immunohistochemical approaches. At 20 M, there were no differences between groups in the total number of microglial cells (Fig. 7b) or astrocytic reactivity (Fig. 7e), measured by GFAP immunofluorescence intensity, in any of the regions of interest including the hippocampus, subiculum (Fig. S6e, i), or entorhinal cortex (Fig. S6f, j). However, microglial cell abundance, as assessed by Iba1 IR, was elevated in the CA1 of 24-month-old Tg rats as compared to Wt rats (Fig. 7c). Notably, microglia appeared to be recruited to the Aβ-burdened neuronal layers of the hippocampus in APP^+/−^ rats, whereas R955-hTau^+/−^ rats uniquely developed the appearance of rod-shaped microglia trailing the apical dendrites of CA1 pyramidal neurons. Furthermore, both these transgene-specific observations were detected in combination in aged APPxhTau rats, implicating a collective synergy in microglia. Signs of astrocytosis also emerged at 24 M as shown by a 2-fold increase in glial fibrillary acidic protein (GFAP) immunofluorescence intensity in APP^+/−^ and R955-hTau^+/−^ as compared to Wt rats (Fig. 7f). Interestingly, in APPxhTau rats, GFAP fluorescence intensity in the CA1 region was greater than that of the single Tg rats and Wt littermates. In addition, increased GFAP fluorescence was detected in other investigated brain regions including the subiculum in 24-month-old R955-hTau^+/−^ and APPxhTau as compared to Wt rats (Fig. S6k). Together, these findings demonstrate a robust and synergistic CNS inflammatory response to the combination of amyloid and tau pathology during the advanced plaque pathology, involving altered glial morphology resembling a reactive state, which may contribute to the accelerated cognitive decline, along with the increased neuronal loss.

## Discussion

Our investigations of the separate pathologies in transgenic models supports several independent studies demonstrating that brain amyloidosis can provoke very early cognitive decline at pre-plaque stages [9, 15, 28, 46, 62, 68, 70, 102, 111]. Such a well-established negative effect of amyloid likely would be unnoticed in the human given its superior cognitive reserve [97, 98, 114]. In comparison, pre-tangle tau pathology as represented in R955-hTau^+/-^ rats has a more subtle impact on cognition than pre-plaque Aβ pathology although it causes initial neuronal loss. However, only the advanced human-like tauopathy, as reproduced in multiple tau transgenic models, does lead to substantive neuronal cell death, brain atrophy and noticeable ventricular dilation [27, 31, 35, 74, 87, 89, 100, 117]. These aspects are particularly pronounced in homozygous R955-hTau rats and in P301S transgenic tau mice crossed to a human ApoE4 background [95].

To further investigate the individual and combined contributions of the AD-like amyloid and tau pathologies to early pathological stages, we utilized the McGill-R-Thy1-APP [71] and the McGill-R955-hTau [35, 36] transgenic rat models to generate the APPxhTau rat line. APPxhTau rats recapitulated features of the earliest disease stages of the amyloid and tau pathologies, providing information which would be difficult, if not impossible, to acquire from human brain samples. These aspects include the initial intraneuronal Aβ accumulation as reported originally in the human brain [45, 112] and reproduced in transgenic mouse [15, 82] and rat models of the amyloid pathology [34, 62]. Such early amyloid burden provokes cognitive deficits in rodents, as also exemplified in APPxhTau rats. The APPxhTau rats also demonstrated to accumulate Sarkosyl-insoluble ptau Thr231, a tau phosphorylated site implicated in early stages of AD [7, 8, 43], without developing overt tau neurofibrillary inclusions (NFT’s). However, the nature of the tau species present in the insoluble fraction – whether they are fibrillar or oligomeric – remains to be established. As expected, at advanced stages the combined overexpression of Aβ and tau had an incremental effect on the classical brain AD-like pathology as well as significant additive effects on cognition, synaptic plasticity, inflammation and neuronal loss. Here we describe a colocalization event between Aβ and human tau in neurons (Fig. 2J). As Aβ and tau have previously been found to colocalize in dystrophic neurites proximal to amyloid plaques [56, 101] and in synaptosomes [37], we suspect that such a colocalization reflects the intraneuronal accumulation of both peptides in early disease stages.

Contrary to our expectations, the present study additionally revealed a transient protective effect of tau against Aβ-induced cognitive impairments at 20 months of age, as illustrated in Fig. 3. These findings do not necessarily contradict contemporary studies that have demonstrated that at late pathological stages such as the transition from MCI to clinical AD, the exponential tau pathology is the main driver of cognitive decline leading to dementia [3, 6, 14, 18, 38, 63, 75, 84]. Interestingly, Morgan and colleagues reported an apparent benefit of overexpressing P301L mutated human tau, driven by the prion promoter, on motor function in the JNPL3 line of APP transgenic mice before the onset of paralysis [78]. The impact of tau on cognition is also dramatically illustrated in an AD individual homozygous for the Christchurch mutation in APOE, which presented low levels of tauopathy and absence of dementia even when displaying an advanced brain amyloidosis [3, 24, 53, 93]. Aβ peptides have a negative effect on LTP formation in ex vivo conditions [105] and such impairment of synaptic function is also observed in vivo [85, 86]. Tau is a cytoskeletal protein that facilitates the stability of microtubules for axonal growth and transport. For these reasons, we hypothesized that the early protective effect on cognition may be mediated by the moderate elevation of tau levels and the likely physiological activities at postsynaptic sites rather than downstream synaptic loss.

Our findings extend and deepen the works of others that have reported a counteractive interaction between Aβ and tau within neuronal circuitry. For example, introducing a human tau transgene was shown to counteract amyloid-induced hyperactivity by suppressing Ca^2+^ currents and neuronal activity in APP/PS1xrTg4510 mice [21]. Similarly, tau dampened the excitatory effects of hAPP on entorhinal circuit excitability in APPxP301L Tg mice as demonstrated in ex vivo electrophysiological analyses [2]. Such a potentially protective effect of tau on cognition might in part explain the decades-long asymptomatic phase during preclinical AD in a scenario of sustained Aβ expression. Certainly, it has long been suspected that the human brain developing AD succumbs to physiological exhaustion through several proposed mechanisms. Our work demonstrates that this counteractive synaptic effect occurs during the early stages of the AD continuum, before the onset of well-characterized events such as extracellular amyloid and neurofibrillary tau deposition. Furthermore, we reasoned that, in these early stages, the physiological activities of tau at the postsynaptic sites might counteract the initial Aβ-induced hyperexcitability, thereby contributing to this distinct early protective effect of tau.

**Fig. 8:**
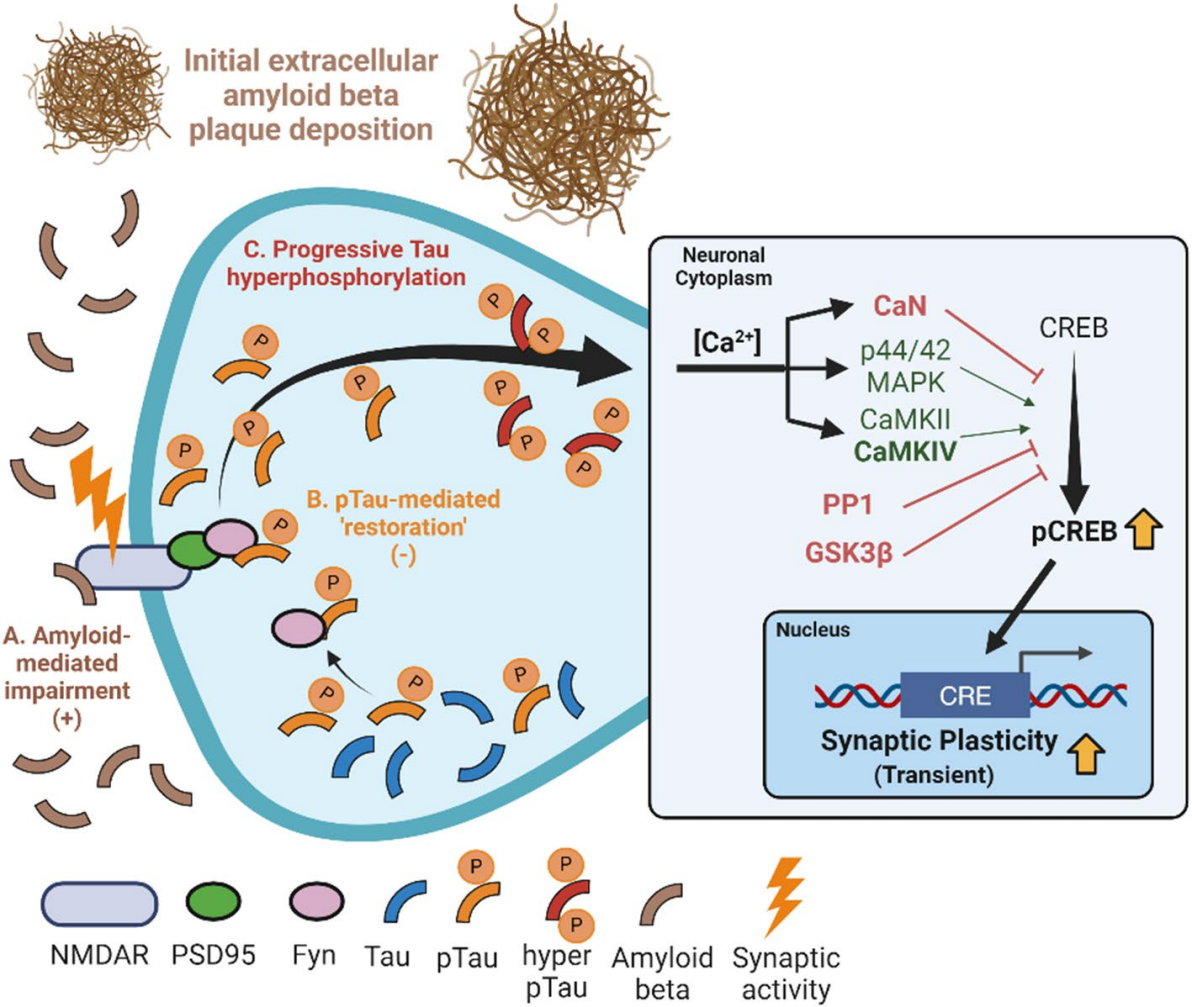
Proposed mechanism of action for the transient effects of tau in early stages of AD-like pathology. **(A)** sufficient Aβ-induced hyperexcitability hinders synaptic function and induces a cascade of cognitive changes. **(B)** tau mediates a temporary stabilization of the NMDAR in a phosphorylation-dependent mechanism, counteracting the effects of Aβ by elevating proteins associated with neuroplasticity signaling such as CREB **(C)** Provided there is no removal of pathology, synaptic tau becomes hyperphosphorylated and aggregation-prone

As illustrated in Fig. 8, we propose a theoretical mechanism for the tau-associated early protective effects. Based on the present study, this mechanism would likely include the increase in dendritic proteins such as Fyn kinase and PSD-95 rendering a neuroprotective phenotype. In line with this, reintroducing Fyn after ablation has been shown to rescue LTP impairments in vivo [20, 25, 59, 64, 81], and the PSD-95 protein has revealed a protective role in synaptic function [33, 65]. Additionally, while postsynaptic PSD-95/tau/Fyn complexes stabilize the NMDAR receptor and mediate Aβ-induced excitotoxicity [60], the site-specific phosphorylation of tau at Thr205 [61] mitigated Aβ toxicity by disrupting NMDAR/PSD-95/tau/Fyn complexes, a consequence consistent with the early appearance and accumulation of ptau Ser202-Thr205 in transgenic rats harbouring the tau transgene. Based on the present findings, we speculate that the early tau-driven protective phenotype might be attributed to increases in Fyn and PSD-95.

In parallel to the increase in Fyn and PSD-95, this study further revealed a perplexing relationship between amyloid and tau on the phosphorylation of CREB Ser133 in a synaptic activity-dependent manner. An abnormal decrease in pCREB and nuclear CRTC1 is affiliated with reduced synaptic plasticity and occurs in AD [5, 76] as well as in animal models of amyloid pathology [110]. Still, the impact of tau on pCREB and CRTC1 has not been fully resolved. Tau accumulation may potentially alter pCREB by activating PP2B and CaMKIV [116]. Therefore, in addition to elevated Fyn and PSD-95, the increased CaMKIV protein expression may contribute to a calcium-dependent restoration of synaptic function in 20-month-old APPxhTau rats, as a sustained CaMKIV activity mediates pCREB at Ser133 and provides neuroprotection from NMDAR-mediated excitotoxicity may further contribute to the elevated pCREB levels. Notably, there was also elevation in GSK3β, which inhibits CREB phosphorylation, but also is intimately tied to tau hyperphosphorylation and excitotoxicity. Our findings further exemplify a dysregulation of GSK3β in AD and is consistent with GSK3β being a well-established link between amyloid and tau [26, 54, 55, 57, 69, 90].

In summary, the slow progressing phenotype of APPxhTau rats has unveiled the existence of an early, yet transient, stage of AD pathology during which tau appears to correct the synaptic dysfunction provoked by the intraneuronal accumulation of Aβ oligomers. Such stage would be difficult to unravel in human brain samples from the earliest preclinical stages of AD and it would be clinically unnoticed in the human given its superior cognitive reserve. However, it might represent a yet unnoticed early component in the continuum of the AD pathology.

## Electronic supplementary material

Below is the link to the electronic supplementary material.

Supplementary Material 1

Supplementary Material 2

Supplementary Material 3

Supplementary Material 4

Supplementary Material 5

Supplementary Material 6

Supplementary Material 7

Supplementary Material 8

Supplementary Material 9

Supplementary Material 10

## Data Availability

No datasets were generated or analysed during the current study.

## References

[1] Allen B, Ingram E, Takao M, Smith MJ, Jakes R, Virdee K, Yoshida H, Holzer M, Craxton M, Emson PC, Atzori C, Migheli A, Crowther RA, Ghetti B, Spillantini MG, Goedert M (2002) Abundant tau filaments and nonapoptotic neurodegeneration in transgenic mice expressing human P301S tau protein. J Neurosci 22:9340–9351. 22/21/9340 [pii] 12417659 10.1523/JNEUROSCI.22-21-09340.2002 PMC6758022

[2] Angulo SL, Orman R, Neymotin SA, Liu L, Buitrago L, Cepeda-Prado E, Stefanov D, Lytton WW, Stewart M, Small SA, Duff KE, Moreno H (2017) Tau and amyloid-related pathologies in the entorhinal cortex have divergent effects in the hippocampal circuit. Neurobiol Dis 108:261–276. 10.1016/j.nbd.2017.08.015 28860088 10.1016/j.nbd.2017.08.015

[3] Arboleda-Velasquez JF, Lopera F, O’Hare M, Delgado-Tirado S, Marino C, Chmielewska N, Saez-Torres KL, Amarnani D, Schultz AP, Sperling RA, Leyton-Cifuentes D, Chen K, Baena A, Aguillon D, Rios-Romenets S, Giraldo M, Guzmán-Vélez E, Norton DJ, Pardilla-Delgado E, Artola A, Sanchez JS, Acosta-Uribe J, Lalli M, Kosik KS, Huentelman MJ, Zetterberg H, Blennow K, Reiman RA, Luo J, Chen Y, Thiyyagura P, Su Y, Jun GR, Naymik M, Gai X, Bootwalla M, Ji J, Shen L, Miller JB, Kim LA, Tariot PN, Johnson KA, Reiman EM, Quiroz YT (2019) Resistance to autosomal dominant Alzheimer’s disease in an APOE3 Christchurch homozygote: a case report. Nat Med 25:1680–1683. 10.1038/s41591-019-0611-3 31686034 10.1038/s41591-019-0611-3 PMC6898984

[4] Arriagada PV, Growdon JH, Hedleywhyte ET, Hyman BT, Hedley-Whyte ET, Hyman BT (1992) Neurofibrillary tangles but not senile plaques parallel duration and severity of alzheimers-Disease. Neurology 42:631–639. 10.1212/WNL.42.3.631 1549228 10.1212/wnl.42.3.631

[5] Arvanitis DN, Ducatenzeiler A, Ou JN, Grodstein E, Andrews SD, Tendulkar SR, Ribeiro-Da-Silva A, Szyf M, Cuello AC (2007) High intracellular concentrations of amyloid-beta block nuclear translocation of phosphorylated CREB. J Neurochem 103:216–228. 10.1111/j.1471-4159.2007.04704.x 17587310 10.1111/j.1471-4159.2007.04704.x

[6] Aschenbrenner AJ, Gordon BA, Benzinger TLS, Morris JC, Hassenstab JJ (2018) Influence of tau PET, amyloid PET, and hippocampal volume on cognition in Alzheimer disease. Neurology 91:e859–e866. 10.1212/WNL.0000000000006075 30068637 10.1212/WNL.0000000000006075 PMC6133625

[7] Ashton NJ, Pascoal TA, Karikari TK, Benedet AL, Lantero-Rodriguez J, Brinkmalm G, Snellman A, Schöll M, Troakes C, Hye A, Gauthier S, Vanmechelen E, Zetterberg H, Rosa-Neto P, Blennow K (2021) Plasma p-tau231: a new biomarker for incipient Alzheimer’s disease pathology. Acta Neuropathol 141:709–724. 10.1007/s00401-021-02275-6 33585983 10.1007/s00401-021-02275-6 PMC8043944

[8] Ashton NJ, Benedet AL, Pascoal TA, Karikari TK, Lantero-Rodriguez J, Brum WS, Mathotaarachchi S, Therriault J, Savard M, Chamoun M, Stoops E, Francois C, Vanmechelen E, Gauthier S, Zimmer ER, Zetterberg H, Blennow K, Rosa-Neto P (2022) Cerebrospinal fluid p-tau231 as an early indicator of emerging pathology in Alzheimer’s disease. EBioMedicine 76:1–13. 10.1016/j.ebiom.2022.103836 10.1016/j.ebiom.2022.103836 PMC8850760 35158308

[9] Bayer TA, Wirths O (2010) Intracellular accumulation of amyloid-beta - A predictor for synaptic dysfunction and neuron loss in Alzheimer’s disease. Front Aging Neurosci 2:1–10. 10.3389/fnagi.2010.00008 20552046 10.3389/fnagi.2010.00008 PMC2879032

[10] Bejanin A, Schonhaut DR, La Joie R, Kramer JH, Baker SL, Sosa N, Ayakta N, Cantwell A, Janabi M, Lauriola M, O’Neil JP, Gorno-Tempini ML, Miller ZA, Rosen HJ, Miller BL, Jagust WJ, Rabinovici GD (2017) Tau pathology and neurodegeneration contribute to cognitive impairment in Alzheimer’s disease. Brain 140:3286–3300. 10.1093/brain/awx243 29053874 10.1093/brain/awx243 PMC5841139

[11] Bell KFS, Ducatenzeiler A, Ribeiro-da-Silva A, Duff K, Bennett DA, Claudio Cuello A (2006) The amyloid pathology progresses in a neurotransmitter-specific manner. Neurobiol Aging 27:1644–1657. 10.1016/j.neurobiolaging.2005.09.034 16271419 10.1016/j.neurobiolaging.2005.09.034

[12] Bell KFS, Bent RJ, Meese-Tamuri S, Ali A, Forder JP, Aarts MM (2013) Calmodulin kinase IV-dependent CREB activation is required for neuroprotection via NMDA receptor-PSD95 disruption. J Neurochem 126:274–287. 10.1111/jnc.12176 23363435 10.1111/jnc.12176

[13] Bennett DA, Schneider JA, Wilson RS, Bienias JL, Arnold SE (2004) Neurofibrillary Tangles Mediate the Association of Amyloid Load with clinical Alzheimer Disease and Level of cognitive function. Arch Neurol 61:378–384. 10.1001/archneur.61.3.378 15023815 10.1001/archneur.61.3.378

[14] Biel D, Brendel M, Rubinski A, Buerger K, Janowitz D, Dichgans M, Franzmeier N (2021) Tau-PET and in vivo braak-staging as prognostic markers of future cognitive decline in cognitively normal to demented individuals. Alzheimers Res Ther 13:1–13. 10.1186/s13195-021-00880-x 34384484 10.1186/s13195-021-00880-x PMC8361801

[15] Billings LM, Oddo S, Green KN, McGaugh JL, LaFerla FM (2005) Intraneuronal Aβ causes the onset of early Alzheimer’s disease-related cognitive deficits in transgenic mice. Neuron 45:675–688. 10.1016/j.neuron.2005.01.040 15748844 10.1016/j.neuron.2005.01.040

[16] Bito H, Deisseroth K, Tsien RW (1996) CREB phosphorylation and dephosphorylation: a Ca2+- and stimulus duration-dependent switch for hippocampal gene expression. Cell 87:1203–1214. 10.1016/S0092-8674(00)81816-4 8980227 10.1016/s0092-8674(00)81816-4

[17] Bloom GS (2014) Amyloid-B and tau: the trigger and bullet in Alzheimer disease pathogenesis. JAMA Neurol 71:505–508. 10.1001/jamaneurol.2013.5847 24493463 10.1001/jamaneurol.2013.5847 PMC12908160

[18] Brier MR, Gordon B, Friedrichsen K, McCarthy J, Stern A, Christensen J, Owen C, Aldea P, Su Y, Hassenstab J, Cairns NJ, Holtzman DM, Fagan AM, Morris JC, Benzinger TLS, Ances BM (2016) Tau and ab imaging, CSF measures, and cognition in Alzheimer’s disease. Sci Transl Med 8:1–9. 10.1126/scitranslmed.aaf2362 10.1126/scitranslmed.aaf2362 PMC5267531 27169802

[19] Brier MR, Gordon B, Morris JH, Benzinger TLS, Ances BM (2016) Tau and Aß imaging, CSF measures, and cognition in Alzheimer’ s disease. Sci Transl Med 8:1–10. 10.1126/scitranslmed.aaf2362 10.1126/scitranslmed.aaf2362 PMC5267531 27169802

[20] Briner A, Götz J, Polanco JC (2020) Fyn kinase controls tau aggregation in vivo. Cell Rep 32. 10.1016/j.celrep.2020.108045 10.1016/j.celrep.2020.108045 32814048

[21] Busche MA, Wegmann S, Dujardin S, Commins C, Schiantarelli J, Klickstein N, Kamath TV, Carlson GA, Nelken I, Hyman BT (2019) Tau impairs neural circuits, dominating amyloid-β effects, in Alzheimer models in vivo. Nat Neurosci 22:57–64. 10.1038/s41593-018-0289-8 30559471 10.1038/s41593-018-0289-8 PMC6560629

[22] Cao W, Zheng H (2018) Correction to: peripheral immune system in aging and Alzheimer’s disease. Mol Neurodegener 13:1–17. 10.1186/s13024-018-0290-4 30355319 10.1186/s13024-018-0290-4 PMC6201538

[23] Chabrier MA, Cheng D, Castello NA, Green KN, LaFerla FM (2014) Synergistic effects of amyloid-beta and wild-type human tau on dendritic spine loss in a floxed double transgenic model of Alzheimer’s disease. Neurobiol Dis 64:107–117. 10.1016/j.nbd.2014.01.007 24440055 10.1016/j.nbd.2014.01.007 PMC4072239

[24] Chen Y, Song S, Parhizkar S, Lord J, Zhu Y, Strickland MR, Wang C, Park J, Tabor GT, Jiang H, Li K, Davis AA, Yuede CM, Colonna M, Ulrich JD, Holtzman DM (2024) APOE3ch alters microglial response and suppresses Aβ-induced tau seeding and spread. Cell 187:428–445e20. 10.1016/j.cell.2023.11.029 38086389 10.1016/j.cell.2023.11.029 PMC10842861

[25] Chin J, Palop JJ, Puoliväli J, Massaro C, Bien-Ly N, Gerstein H, Scearce-Levie K, Masliah E, Mucke L (2005) Fyn kinase induces synaptic and cognitive impairments in a transgenic mouse model of Alzheimer’s disease. J Neurosci 25:9694–9703. 10.1523/JNEUROSCI.2980-05.2005 16237174 10.1523/JNEUROSCI.2980-05.2005 PMC6725734

[26] Cho J, Johnson GVW (2004) Primed phosphorylation of tau at Thr231 by glycogen synthase kinase 3B (GSK3B) plays a critical role in regulating tau’s ability to bind and stabilize microtubules. J Neurochem 88:349–358. 10.1046/j.1471-4159.2003.02155.x 14690523 10.1111/j.1471-4159.2004.02155.x

[27] Cohen RM, Rezai-zadeh K, Weitz TM, Rentsendorj A, Gate D, Spivak I, Bholat Y, Vasilevko V, Glabe CG, Breunig JJ, Rakic P, Davtyan H, Agadjanyan MG (2013) A transgenic Alzheimer rat with plaques, tau pathology, behavioral impairment, oligomeric Aβ and frank neuronal loss. J Neurosci 33:6245–6256. 10.1523/JNEUROSCI.3672-12.2013.A 23575824 10.1523/JNEUROSCI.3672-12.2013 PMC3720142

[28] Cuello AC, Ferretti MT, Iulita MF (2012) Preplaque (‘preclinical’) Aβ-induced inflammation and nerve growth factor deregulation in transgenic models of Alzheimer’s disease-like amyloid pathology. Neurodegener Dis 10:104–107. 10.1159/000333339 22261363 10.1159/000333339

[29] Cummings J, Lee G, Nahed P, Kambar MEZN, Zhong K, Fonseca J, Taghva K (2022) Alzheimer’s disease drug development pipeline: 2022. Alzheimer’s and Dementia: Translational Research and clinical interventions 8. 10.1002/trc2.12295 10.1002/trc2.12295 PMC9066743 35516416

[30] Deisseroth K, Bito H, Tsien RW (1996) Signaling from synapse to nucleus: postsynaptic CREB phosphorylation during multiple forms of hippocampal synaptic plasticity. Neuron 16:89–101. 10.1016/S0896-6273(00)80026-4 8562094 10.1016/s0896-6273(00)80026-4

[31] Di J, Cohen LS, Corbo CP, Phillips GR, El Idrissi A, Alonso AD (2016) Abnormal tau induces cognitive impairment through two different mechanisms: synaptic dysfunction and neuronal loss. Sci Rep 6:1–12. 10.1038/srep20833 26888634 10.1038/srep20833 PMC4757872

[32] Do Carmo S, Cuello AC (2013) Modeling Alzheimer’s disease in transgenic rats. Mol Neurodegener 8:37. 10.1186/1750-1326-8-37 24161192 10.1186/1750-1326-8-37 PMC4231465

[33] Dore K, Carrico Z, Alfonso S, Marino M, Koymans K, Kessels HW, Malinow R (2021) PSD-95 protects synapses from β-amyloid. Cell Rep 35:109194. 10.1016/j.celrep.2021.109194 34077732 10.1016/j.celrep.2021.109194 PMC8237704

[34] Echeverria V, Ducatenzeiler A, Dowd E, Jänne J, Grant SM, Szyf M, Wandosell F, Avila J, Grimm H, Dunnett SB, Hartmann T, Alhonen L, Cuello AC (2004) Altered mitogen-activated protein kinase signaling, tau hyperphosphorylation and mild spatial learning dysfunction in transgenic rats expressing the β-amyloid peptide intracellularly in hippocampal and cortical neurons. Neuroscience 129:583–592. 10.1016/j.neuroscience.2004.07.036 15541880 10.1016/j.neuroscience.2004.07.036

[35] Emmerson JT, Do Carmo S, Liu Y, Shalhoub A, Liu A, Bonomo Q, Malcolm JC, Breuillaud L, Cuello AC (2023) Progressive human-like tauopathy with downstream neurodegeneration and neurovascular compromise in a transgenic rat model. Neurobiol Dis 184:106227. 10.1016/j.nbd.2023.106227 37454780 10.1016/j.nbd.2023.106227

[36] Emmerson JT, Malcolm JC, Do Carmo S, Nguyen P, Breuillaud L, Martinez-Trujillo JC, Cuello AC (2023) Neuronal loss and inflammation preceding fibrillary tau pathology in a rat model with early human-like tauopathy. Neurobiol Dis 187:106317. 10.1016/j.nbd.2023.106317 37802153 10.1016/j.nbd.2023.106317

[37] Fein JA, Sokolow S, Miller CA, Vinters HV, Yang F, Cole GM, Gylys KH (2008) Co-localization of amyloid beta and tau pathology in Alzheimer’s disease synaptosomes. Am J Pathol 172:1683–1692. 10.2353/ajpath.2008.070829 18467692 10.2353/ajpath.2008.070829 PMC2408427

[38] Ferreira A, Lu Q, Orecchio L, Kosik KS (1997) Selective phosphorylation of adult tau isoforms in mature hippocampal neurons exposed to fibrillar aβ. Mol Cell Neurosci 9:220–234. 10.1006/mcne.1997.0615 9245504 10.1006/mcne.1997.0615

[39] Florian H, Wang D, Arnold SE, Boada M, Guo Q, Jin Z, Zheng H, Fisseha N, Kalluri HV, Rendenbach-Mueller B, Budur K, Gold M (2023) Tilavonemab in early Alzheimer’s disease: results from a phase 2, randomized, double-blind study. Brain. 10.1093/brain/awad024 36730056 10.1093/brain/awad024 PMC10232284

[40] Fortea J, Vilaplana E, Alcolea D, Carmona-Iragui M, Sánchez-Saudinos MB, Sala I, Antón-Aguirre S, González S, Medrano S, Pegueroles J, Morenas E, Clarimón J, Blesa R, Lleó A (2014) Cerebrospinal fluid β-amyloid and phospho-tau biomarker interactions affecting brain structure in preclinical Alzheimer disease. Ann Neurol 76:223–230. 10.1002/ana.24186 24852682 10.1002/ana.24186

[41] Galeano P, Martino Adami PV, Do Carmo S, Blanco E, Rotondaro C, Capani F, Castaño EM, Cuello AC, Morelli L (2014) Longitudinal analysis of the behavioral phenotype in a novel transgenic rat model of early stages of Alzheimer’s disease. Front Behav Neurosci 8:1–15. 10.3389/fnbeh.2014.00321 25278855 10.3389/fnbeh.2014.00321 PMC4165352

[42] Giannakopoulos P, Herrmann FR, Bussière T, Bouras C, Kövari E, Perl DP, Morrison JH, Gold G, Hof PR (2003) Tangle and neuron numbers, but not amyloid load, predict cognitive status in Alzheimer’s disease. Neurology 60:1495–1500. 10.1212/01.WNL.0000063311.58879.01 12743238 10.1212/01.wnl.0000063311.58879.01

[43] Glodzik L, de Santi S, Tsui WH, Mosconi L, Zinkowski R, Pirraglia E, Wang HY, Li Y, Rich KE, Zetterberg H, Blennow K, Mehta P, de Leon MJ (2011) Phosphorylated tau 231, memory decline and medial temporal atrophy in normal elders. Neurobiol Aging 32:2131–2141. 10.1016/j.neurobiolaging.2009.12.026 20133017 10.1016/j.neurobiolaging.2009.12.026 PMC3179835

[44] Gotz J, Chen F, van Dorpe J, Nitsch RM (2001) Formation of Neurofibrillary Tangles in P301L Tau Transgenic Mice Induced by Abeta 42 Fibrils. Science (1979) 293:1491–1495. 10.1126/science.1062097 10.1126/science.1062097 11520988

[45] Gouras GK, Tsai J, Naslund J, Vincent B, Edgar M, Checler F, Greenfield JP, Haroutunian V, Buxbaum JD, Xu H, Greengard P, Relkin NR (2000) Intraneuronal Aβ42 accumulation in human brain. Am J Pathol 156:15–20. 10.1016/S0002-9440(10)64700-1 10623648 10.1016/s0002-9440(10)64700-1 PMC1868613

[46] Gouras GK, Almeida CG, Takahashi RH (2005) Intraneuronal Aβ accumulation and origin of plaques in Alzheimer’s disease. Neurobiol Aging 26:1235–1244. 10.1016/j.neurobiolaging.2005.05.022 16023263 10.1016/j.neurobiolaging.2005.05.022

[47] Grant SM, Ducatenzeiler A, Szyf M, Cuello AC (2000) Abeta immunoreactive material is present in several intracellular compartments in transfected, neuronally differentiated, P19 cells expressing the human amyloid beta-protein precursor. J Alzheimers Dis 2:207–222 12214085 10.3233/jad-2000-23-403

[48] Guillozet AL, Weintraub S, Mash DC, Marsel Mesulam M (2003) Neurofibrillary tangles, amyloid, and memory in aging and mild cognitive impairment. Arch Neurol 60:729–736. 10.1001/archneur.60.5.729 12756137 10.1001/archneur.60.5.729

[49] Hall H, Iulita MF, Gubert P, Flores Aguilar L, Ducatenzeiler A, Fisher A, Cuello AC (2018) AF710B, an M1/sigma-1 receptor agonist with long-lasting disease-modifying properties in a transgenic rat model of Alzheimer’s disease. Alzheimer’s Dement 14:811–823. 10.1016/j.jalz.2017.11.009 29291374 10.1016/j.jalz.2017.11.009

[50] Hanes J, Zilka N, Bartkova M, Caletkova M, Dobrota D, Novak M (2009) Rat tau proteome consists of six tau isoforms: implication for animal models of human tauopathies. J Neurochem 108:1167–1176. 10.1111/j.1471-4159.2009.05869.x 19141083 10.1111/j.1471-4159.2009.05869.x

[51] Hanseeuw BJ, Betensky RA, Jacobs HIL, Schultz AP, Sepulcre J, Becker JA, Cosio DMO, Farrell M, Quiroz YT, Mormino EC, Buckley RF, Papp KV, Amariglio RA, Dewachter I, Ivanoiu A, Huijbers W, Hedden T, Marshall GA, Chhatwal JP, Rentz DM, Sperling RA, Johnson K (2019) Association of Amyloid and tau with cognition in preclinical Alzheimer Disease: a longitudinal study. JAMA Neurol 76:915–924. 10.1001/jamaneurol.2019.1424 31157827 10.1001/jamaneurol.2019.1424 PMC6547132

[52] Hanzel CE, Pichet-Binette A, Pimentel LSB, Iulita MF, Allard S, Ducatenzeiler A, Do Carmo S, Cuello AC (2014) Neuronal driven pre-plaque inflammation in a transgenic rat model of Alzheimer’s disease. Neurobiol Aging 35:2249–2262. 10.1016/j.neurobiolaging.2014.03.026 24831823 10.1016/j.neurobiolaging.2014.03.026

[53] Henao-Restrepo J, López-Murillo C, Valderrama-Carmona P, Orozco-Santa N, Gomez J, Gutiérrez-Vargas J, Moraga R, Toledo J, Littau JL, Härtel S, Arboleda-Velásquez JF, Sepulveda-Falla D, Lopera F, Cardona-Gómez GP, Villegas A, Posada-Duque R (2023) Gliovascular alterations in sporadic and familial Alzheimer’s disease: APOE3 Christchurch homozygote glioprotection. Brain Pathol 33:1–23. 10.1111/bpa.13119 10.1111/bpa.13119 PMC10041169 36130084

[54] Hernandez F, Lucas JJ, Avila J (2012) GSK3 and tau: two convergence points in Alzheimer’s Disease. J Alzheimer’s Disease 33:S141–S144. 10.3233/JAD-2012-129025 10.3233/JAD-2012-129025 22710914

[55] Hernández F, Gómez de Barreda E, Fuster-Matanzo A, Lucas JJ, Avila J (2010) GSK3: a possible link between beta amyloid peptide and tau protein. Exp Neurol 223:322–325. 10.1016/j.expneurol.2009.09.011 19782073 10.1016/j.expneurol.2009.09.011

[56] Hirota Y, Sakakibara Y, Ibaraki K, Takei K, Iijima KM, Sekiya M (2022) Distinct brain pathologies associated with Alzheimer’s disease biomarker-related phospho-tau 181 and phospho-tau 217 in app knock-in mouse models of amyloid-β amyloidosis. Brain Commun 4:1–15. 10.1093/braincomms/fcac286 10.1093/braincomms/fcac286 PMC9683396 36440096

[57] Hooper C, Killick R, Lovestone S (2008) The GSK3 hypothesis of Alzheimer’s disease. J Neurochem 104:1433–1439. 10.1111/j.1471-4159.2007.05194.x 18088381 10.1111/j.1471-4159.2007.05194.x PMC3073119

[58] Hu NW, Smith IM, Walsh DM, Rowan MJ (2008) Soluble amyloid-β peptides potently disrupt hippocampal synaptic plasticity in the absence of cerebrovascular dysfunction in vivo. Brain 131:2414–2424. 10.1093/brain/awn174 18678563 10.1093/brain/awn174

[59] Iannuzzi F, Sirabella R, Canu N, Maier TJ, Annunziato L, Matrone C (2020) Fyn tyrosine kinase elicits amyloid precursor protein Tyr682 phosphorylation in neurons from Alzheimer’s Disease patients. Cells 9:1–21. 10.3390/cells9081807 10.3390/cells9081807 PMC7463977 32751526

[60] Ittner LM, Ke YD, Delerue F, Bi M, Gladbach A, van Eersel J, Wölfing H, Chieng BC, Christie MJ, Napier IA, Eckert A, Staufenbiel M, Hardeman E, Götz J (2010) Dendritic function of tau mediates amyloid-β toxicity in alzheimer’s disease mouse models. Cell 142:387–397. 10.1016/j.cell.2010.06.036 20655099 10.1016/j.cell.2010.06.036

[61] Ittner A, Chua SW, Bertz J, Volkerling A, van der Hoven J, Gladbach A, Przybyla M, Bi M, van Hummel A, Stevens CH, Ippati S, Suh LS, Macmillan A, Sutherland G, Kril JJ, Silva APG, Mackay JP, Poljak A, Delerue F, Ke YD, Ittner LM (2016) Site-specific phosphorylation of tau inhibits amyloid-β toxicity in Alzheimer’s mice. Science (1979) 354:904–908. 10.1126/science.aah6205 10.1126/science.aah6205 27856911

[62] Iulita MF, Allard S, Richter L, Munter L-M, Ducatenzeiler A, Weise C, Do Carmo S, Klein WL, Multhaup G, Cuello AC (2014) Intracellular Aβ pathology and early cognitive impairments in a transgenic rat overexpressing human amyloid precursor protein: a multidimensional study. Acta Neuropathol Commun 2:61. 10.1186/2051-5960-2-61 24903713 10.1186/2051-5960-2-61 PMC4229908

[63] Jack CR (2020) Predicting future rates of tau accumulation on PET. Alzheimer’s Dement 16:44594. 10.1002/alz.044594 10.1093/brain/awaa248 PMC7586089 33094327

[64] Kojima N, Wang J, Mansu IM, Grant SGN, Mayford M, Kandel ER (1997) Rescuing impairment of long-term potentiation in fyn-deficient mice by introducing Fyn transgene. Proc Natl Acad Sci U S A 94:4761–4765. 10.1073/pnas.94.9.4761 9114065 10.1073/pnas.94.9.4761 PMC20798

[65] Kornau HC, Kornau HC, Schenker LT, Kennedy MB, Seeburg PH (1995) Domain interaction between NMDA receptor subunits and the postsynaptic density protein PSD-95. Science (1979) 1737:1737–1740. 10.1126/science.7569905 10.1126/science.7569905 7569905

[66] Krüger L, Mandelkow EM (2016) Tau neurotoxicity and rescue in animal models of human tauopathies. Curr Opin Neurobiol 36:52–58. 10.1016/j.conb.2015.09.004 26431808 10.1016/j.conb.2015.09.004

[67] Kummer KK, Hofhansel L, Barwitz CM, Schardl A, Prast JM, Salti A, El Rawas R, Zernig G (2014) Differences in social interaction- vs. cocaine reward in mouse vs. rat. Front Behav Neurosci 8:1–7. 10.3389/fnbeh.2014.00363 25368560 10.3389/fnbeh.2014.00363 PMC4201146

[68] LaFerla FM, Green KN, Oddo S (2007) Intracellular amyloid-β in Alzheimer’s disease. Nat Rev Neurosci 8:499–509. 10.1038/nrn2168 17551515 10.1038/nrn2168

[69] Lauretti E, Dincer O, Praticò D (2020) Glycogen synthase kinase-3 signaling in Alzheimer’s disease. Biochim Biophys Acta Mol Cell Res 1867:118664. 10.1016/j.bbamcr.2020.118664 32006534 10.1016/j.bbamcr.2020.118664 PMC7047718

[70] Lauritzen I, Pardossi-Piquard R, Bauer C, Brigham E, Abraham JD, Ranaldi S, Fraser P, St-George-Hyslop P, Le Thuc O, Espin V, Chami L, Dunys J, Checler F (2012) The β-secretase-derived C-terminal fragment of βAPP, C99, but not Aβ, is a key contributor to early intraneuronal lesions in triple-transgenic mouse hippocampus. J Neurosci 32:16243–16255. 10.1523/JNEUROSCI.2775-12.2012 23152608 10.1523/JNEUROSCI.2775-12.2012 PMC5019353

[71] Leon WC, Canneva F, Partridge V, Allard S, Ferretti MT, Dewilde A, Vercauteren F, Atifeh R, Ducatenzeiler A, Klein W, Szyf M, Alhonen L, Cuello AC (2010) A novel transgenic rat model with a full Alzheimer’s-Like amyloid pathology displays pre-plaque intracellular amyloid-B-Associated cognitive impairment. J Alzheimer’s Disease 20:113–126. 10.3233/JAD-2010-1349 20164597 10.3233/JAD-2010-1349

[72] Leung C, Kim J, Jia Z (2018) Three-chamber Social Approach Task with Optogenetic Stimulation (mice). Bio Protoc 8:1–12. 10.21769/bioprotoc.3120 10.21769/BioProtoc.3120 PMC8342085 34532561

[73] Lewis J, Dickson DW, Lin W, Chisholm L, Corral A, Jones G, Yen S, Sahara N, Skipper L, Yager D, Eckman C, Hutton M, Mcgowan E, Hardy J (2001) Neurofibrillary Degeneration in Transgenic Mice Expressing Mutant tau and APP. Science (1979) 293:1487–1491 10.1126/science.1058189 11520987

[74] Malcolm JC, Breuillaud L, Do Carmo S, Hall H, Welikovitch LA, Macdonald JA, Goedert M, Cuello AC (2019) Neuropathological changes and cognitive deficits in rats transgenic for human mutant tau recapitulate human tauopathy. Neurobiol Dis 127:323–338. 10.1016/j.nbd.2019.03.018 30905766 10.1016/j.nbd.2019.03.018 PMC6597947

[75] McDade E, Wang G, Gordon BA, Hassenstab J, Benzinger TLS, Buckles V, Fagan AM, Holtzman DM, Cairns NJ, Goate AM, Marcus DS, Morris JC, Paumier K, Xiong C, Allegri R, Berman SB, Klunk W, Noble J, Ringman J, Ghetti B, Farlow M, Sperling RA, Chhatwal J, Salloway S, Graff-Radford NR, Schofield PR, Masters C, Rossor MN, Fox NC, Levin J, Jucker M, Bateman RJ (2018) Longitudinal cognitive and biomarker changes in dominantly inherited Alzheimer disease. Neurology 91:E1295–E1306. 10.1212/WNL.0000000000006277 30217935 10.1212/WNL.0000000000006277 PMC6177272

[76] Mendioroz M, Celarain N, Altuna M, Sánchez-Ruiz De Gordoa J, Zelaya MV, Roldán M, Rubio I, Larumbe R, Erro ME, Méndez I, Echávarri C (2016) CRTC1 gene is differentially methylated in the human hippocampus in Alzheimer’s disease. Alzheimers Res Ther 8:1–9. 10.1186/s13195-016-0183-0 27094739 10.1186/s13195-016-0183-0 PMC4837517

[77] Mintun MA, Lo AC, Duggan Evans C, Wessels AM, Ardayfio PA, Andersen SW, Shcherbinin S, Sparks J, Sims JR, Brys M, Apostolova LG, Salloway SP, Skovronsky DM (2021) Donanemab in Early Alzheimer’s Disease. N Engl J Med 384:1691–1704. 10.1056/NEJMoa2100708 33720637 10.1056/NEJMoa2100708

[78] Morgan D, Munireddy S, Alamed J, DeLeon J, Diamond DM, Bickford P, Hutton M, Lewis J, McGowan E, Gordon MN (2011) Apparent behavioral benefits of tau overexpression in P301L tau transgenic mice. Adv Alzheimer Dis 1:129–138. 10.3233/978-1-60750-733-8-129 10.3233/jad-2008-15407 PMC3018352 19096159

[79] Mucke L, Selkoe DJ (2012) Neurotoxicity of amyloid -protein: synaptic and network dysfunction. Cold Spring Harb Perspect Med 2:a006338–a006338. 10.1101/cshperspect.a006338 22762015 10.1101/cshperspect.a006338 PMC3385944

[80] Novak P, Kovacech B, Katina S, Schmidt R, Scheltens P, Kontsekova E, Ropele S, Fialova L, Kramberger M, Paulenka-Ivanovova N, Smisek M, Hanes J, Stevens E, Kovac A, Sutovsky S, Parrak V, Koson P, Prcina M, Galba J, Cente M, Hromadka T, Filipcik P, Piestansky J, Samcova M, Prenn-Gologranc C, Sivak R, Froelich L, Fresser M, Rakusa M, Harrison J, Hort J, Otto M, Tosun D, Ondrus M, Winblad B, Novak M, Zilka N (2021) ADAMANT: a placebo-controlled randomized phase 2 study of AADvac1, an active immunotherapy against pathological tau in Alzheimer’s disease. Nat Aging 1:521–534. 10.1038/s43587-021-00070-2 37117834 10.1038/s43587-021-00070-2

[81] Nygaard HB, Dyck CH, Van, Strittmatter SM (2014) Fyn kinase inhibition as a novel therapy for AD. Alzheimers Res Ther 6:1–8 24495408 10.1186/alzrt238 PMC3978417

[82] Oddo S, Caccamo A, Shepherd JD, Murphy MP, Golde TE, Kayed R, Metherate R, Mattson MP, Akbari Y, LaFerla FM (2003) Triple-transgenic model of Alzheimer’s Disease with plaques and tangles: intracellular Aβ and synaptic dysfunction. Neuron 39:409–421. 10.1016/S0896-6273(03)00434-3 12895417 10.1016/s0896-6273(03)00434-3

[83] Pampuscenko K, Morkuniene R, Krasauskas L, Smirnovas V, Tomita T, Borutaite V (2021) Distinct neurotoxic effects of Extracellular Tau species in primary neuronal-glial cultures. Mol Neurobiol 58:658–667. 10.1007/s12035-020-02150-7 33001416 10.1007/s12035-020-02150-7

[84] Pontecorvo MJ, Devous MD, Kennedy I, Navitsky M, Lu M, Galante N, Salloway S, Murali Doraiswamy P, Southekal S, Arora AK, McGeehan A, Lim NC, Xiong H, Truocchio SP, Joshi AD, Shcherbinin S, Teske B, Fleisher AS, Mintun MA (2019) A multicentre longitudinal study of flortaucipir (18F) in normal ageing, mild cognitive impairment and Alzheimer’s disease dementia. Brain 142:1723–1735. 10.1093/brain/awz090 31009046 10.1093/brain/awz090 PMC6536847

[85] Qi Y, Klyubin I, Harney SC, Hu N, Cullen WK, Grant MK, Steffen J, Wilson EN, Do Carmo S, Remy S, Fuhrmann M, Ashe KH, Cuello AC, Rowan MJ (2014) Longitudinal testing of hippocampal plasticity reveals the onset and maintenance of endogenous human Aß-induced synaptic dysfunction in individual freely behaving pre-plaque transgenic rats: rapid reversal by anti-Aß agents. Acta Neuropathol Commun 2:175. 10.1186/s40478-014-0175-x 25540024 10.1186/s40478-014-0175-x PMC4293804

[86] Qi Y, Klyubin I, Hu NW, Ondrejcak T, Rowan MJ (2019) Pre-plaque Aß-Mediated impairment of synaptic depotentiation in a transgenic rat model of Alzheimer’s Disease Amyloidosis. Front Neurosci 13:1–13. 10.3389/fnins.2019.00861 31474823 10.3389/fnins.2019.00861 PMC6702302

[87] Ramsden M, Kotilinek L, Forster C, Paulson J, McGowan E, SantaCruz K, Guimaraes A, Yue M, Lewis J, Carlson G, Hutton M, Ashe KH (2005) Age-dependent neurofibrillary tangle formation, neuron loss, and memory impairment in a mouse model of human tauopathy (P301L). J Neurosci 25:10637–10647. 10.1523/JNEUROSCI.3279-05.2005 16291936 10.1523/JNEUROSCI.3279-05.2005 PMC6725849

[88] Sakamoto K, Karelina K, Obrietan K (2011) CREB: a multifaceted regulator of neuronal plasticity and protection. J Neurochem 116:1–9. 10.1111/j.1471-4159.2010.07080.x 21044077 10.1111/j.1471-4159.2010.07080.x PMC3575743

[89] Saul A, Sprenger F, Bayer TA, Wirths O (2013) Accelerated tau pathology with synaptic and neuronal loss in a novel triple transgenic mouse model of Alzheimer’s disease. Neurobiol Aging 34:2564–2573. 10.1016/j.neurobiolaging.2013.05.003 23747045 10.1016/j.neurobiolaging.2013.05.003

[90] Schaffer BAJ, Bertram L, Miller BL, Mullin K, Weintraub S, Johnson N, Bigio EH, Mesulam M, Wiedau-Pazos M, Jackson GR, Cummings JL, Cantor RM, Levey AI, Tanzi RE, Geschwind DH (2008) Association of GSK3B with Alzheimer Disease and Frontotemporal Dementia. Arch Neurol 65:1368–1374. 10.1001/archneur.65.10.1368 18852354 10.1001/archneur.65.10.1368 PMC2841136

[91] Schöll M, Lockhart SN, Schonhaut DR, O’Neil JP, Janabi M, Ossenkoppele R, Baker SL, Vogel JW, Faria J, Schwimmer HD, Rabinovici GD, Jagust WJ (2016) PET imaging of tau deposition in the Aging Human Brain. Neuron 89:971–982. 10.1016/j.neuron.2016.01.028 26938442 10.1016/j.neuron.2016.01.028 PMC4779187

[92] Sebastián-Serrano Á, de Diego-García L, Díaz-Hernández M (2018) The neurotoxic role of Extracellular tau protein. Int J Mol Sci 19:998. 10.3390/ijms19040998 29584657 10.3390/ijms19040998 PMC5979432

[93] Sepulveda-Falla D, Sanchez JS, Almeida MC, Boassa D, Acosta-Uribe J, Vila-Castelar C, Ramirez-Gomez L, Baena A, Aguillon D, Villalba-Moreno ND, Littau JL, Villegas A, Beach TG, White CL, Ellisman M, Krasemann S, Glatzel M, Johnson KA, Sperling RA, Reiman EM, Arboleda-Velasquez JF, Kosik KS, Lopera F, Quiroz YT (2022) Distinct tau neuropathology and cellular profiles of an APOE3 Christchurch homozygote protected against autosomal dominant Alzheimer’s dementia. Acta Neuropathol 144:589–601. 10.1007/s00401-022-02467-8 35838824 10.1007/s00401-022-02467-8 PMC9381462

[94] Sevigny J, Chiao P, Bussière T, Weinreb PH, Williams L, Maier M, Dunstan R, Salloway S, Chen T, Ling Y, O’Gorman J, Qian F, Arastu M, Li M, Chollate S, Brennan MS, Quintero-Monzon O, Scannevin RH, Arnold HM, Engber T, Rhodes K, Ferrero J, Hang Y, Mikulskis A, Grimm J, Hock C, Nitsch RM, Sandrock A (2016) The antibody aducanumab reduces Aβ plaques in Alzheimer’s disease. Nature 537:50–56. 10.1038/nature19323 27582220 10.1038/nature19323

[95] Shi Y, Yamada K, Liddelow SA, Smith ST, Zhao L, Luo W, Tsai RM, Spina S, Grinberg LT, Rojas JC, Gallardo G, Wang K, Roh J, Robinson G, Finn MB, Jiang H, Sullivan PM, Baufeld C, Wood MW, Sutphen C, McCue L, Xiong C, Del-Aguila JL, Morris JC, Cruchaga C, Fagan AM, Miller BL, Boxer AL, Seeley WW, Butovsky O, Barres BA, Paul SM, Holtzman DM (2017) ApoE4 markedly exacerbates tau-mediated neurodegeneration in a mouse model of tauopathy. Nature 549:523–527. 10.1038/nature24016 28959956 10.1038/nature24016 PMC5641217

[96] Sosulina L, Mittag M, Geis H-R, Hoffmann K, Klyubin I, Qi Y, Steffen J, Friedrichs D, Henneberg N, Fuhrmann F, Justus D, Keppler K, Cuello AC, Rowan M, Fuhrmann M, Remy S (2020) Hippocampal hyperactivity in a rat model of Alzheimer’s disease. 10.1101/2020.06.09.141598 10.1111/jnc.15323 33583024

[97] Stern Y (2002) What is cognitive reserve? Theory and research application of the reserve concept. J Int Neuropsychol Soc 8:448–460 11939702

[98] Stern Y, Arenaza-Urquijo EM, Bartrés-Faz D, Belleville S, Cantilon M, Chetelat G, Ewers M, Franzmeier N, Kempermann G, Kremen WS, Okonkwo O, Scarmeas N, Soldan A, Udeh-Momoh C, Valenzuela M, Vemuri P, Vuoksimaa E, Urquiljo EMA, Cantillon M, Clouston SAP, Estanga A, Gold B, Habeck C, Jones R, Kochhann R, Lim YY, Martínez-Lage P, Morbelli S, Okonkwo O, Ossenkoppele R, Pettigrew C, Rosen AC, Song X, Van Loenhoud AC (2020) Whitepaper: defining and investigating cognitive reserve, brain reserve, and brain maintenance. Alzheimer’s Dement 16:1305–1311. 10.1016/j.jalz.2018.07.219 30222945 10.1016/j.jalz.2018.07.219 PMC6417987

[99] Strozyk D, Blennow K, White LR, Launer LJ (2003) CSF Aß 42 levels correlate with amyloid-neuropathology in a population-based autopsy study. Neurology 60:652–656. 10.1212/01.WNL.0000046581.81650.D0 12601108 10.1212/01.wnl.0000046581.81650.d0

[100] Sydow A, Van Der Jeugd A, Zheng F, Ahmed T, Balschun D, Petrova O, Drexler D, Zhou L, Rune G, Mandelkow E, D’Hooge R, Alzheimer C, Mandelkow EM (2011) Tau-induced defects in synaptic plasticity, learning, and memory are reversible in transgenic mice after switching off the toxic tau mutant. J Neurosci 31:2511–2525. 10.1523/JNEUROSCI.5245-10.2011 21325519 10.1523/JNEUROSCI.5245-10.2011 PMC6623704

[101] Takahashi RH, Capetillo-Zarate E, Lin MT, Milner TA, Gouras GK (2010) Co-occurrence of Alzheimer’s disease β-amyloid and tau pathologies at synapses. Neurobiol Aging 31:1145–1152. 10.1016/j.neurobiolaging.2008.07.021 18771816 10.1016/j.neurobiolaging.2008.07.021 PMC2909664

[102] Tampellini D, Capetillo-Zarate E, Dumont M, Huang Z, Yu F, Lin MT, Gouras GK (2010) Effects of synaptic modulation on β-amyloid, synaptophysin, and memory performance in Alzheimer’s disease transgenic mice. J Neurosci 30:14299–14304. 10.1523/JNEUROSCI.3383-10.2010 20980585 10.1523/JNEUROSCI.3383-10.2010 PMC2972675

[103] Tran TN, Kim SH, Gallo C, Amaya M, Kyees J, Narayanaswami V (2013) Biochemical and biophysical characterization of recombinant rat apolipoprotein E: similarities to human apolipoprotein E3. Arch Biochem Biophys 529:18–25. 10.1016/j.abb.2012.10.007 23103361 10.1016/j.abb.2012.10.007 PMC3543869

[104] Vergara C, Houben S, Suain V, Yilmaz Z, De Decker R, Vanden Dries V, Boom A, Mansour S, Leroy K, Ando K, Brion J-P (2019) Amyloid-β pathology enhances pathological fibrillary tau seeding induced by Alzheimer PHF in vivo. Acta Neuropathol 137:397–412. 10.1007/s00401-018-1953-5 30599077 10.1007/s00401-018-1953-5

[105] Walsh DM, Klyubin I, Fadeeva JV, Cullen WK, Anwyl R, Wolfe MS, Rowan MJ, Selkoe DJ (2002) Naturally secreted oligomers of amyloid β protein potently inhibit hippocampal long-term potentiation in vivo. Nature 416:535–539. 10.1038/416535a 11932745 10.1038/416535a

[106] Ward SM, Himmelstein DS, Lancia JK, Binder LI (2012) Tau oligomers and tau toxicity in neurodegenerative disease. Biochem Soc Trans 40:667–671. 10.1042/BST20120134 22817713 10.1042/BST20120134 PMC3704193

[107] Whishaw IQ, Tomie J (1996) Of mice and mazes: similarities between mice and rats on Dry Land but Not Water mazes. Physiol Behav 60:1191–1197. 10.1016/S0031-9384(96)00176-X 8916170 10.1016/s0031-9384(96)00176-x

[108] Wilcock GK, Gauthier S, Frisoni GB, Jia J, Hardlund JH, Moebius HJ, Bentham P, Kook KA, Schelter BO, Wischik DJ, Davis CS, Staff RT, Vuksanovic V, Ahearn T, Bracoud L, Shamsi K, Marek K, Seibyl J, Riedel G, Storey JMD, Harrington CR, Wischik CM (2018) Potential of Low Dose Leuco-Methylthioninium Bis(Hydromethanesulphonate) (LMTM) Monotherapy for treatment of mild Alzheimer’s Disease: Cohort Analysis as Modified Primary Outcome in a phase III clinical trial. J Alzheimer’s Disease 61:435–457. 10.3233/JAD-170560 29154277 10.3233/JAD-170560 PMC5734125

[109] Wildner G (2019) Are rats more human than mice? Immunobiology 224:172–176. 10.1016/j.imbio.2018.09.002 30342883 10.1016/j.imbio.2018.09.002

[110] Wilson EN, Abela AR, Do Carmo S, Allard S, Marks AR, Welikovitch LA, Ducatenzeiler A, Chudasama Y, Cuello AC (2016) Intraneuronal amyloid Beta Accumulation disrupts hippocampal CRTC1-Dependent gene expression and cognitive function in a rat model of Alzheimer Disease. Cereb Cortex 27:1–11. 10.1093/cercor/bhv332 10.1093/cercor/bhv332 PMC5378482 26759481

[111] Wirths O, Multhaup G, Czech C, Blanchard V, Moussaoui S, Tremp G, Pradier L, Beyreuther K, Bayer TA (2001) Intraneuronal Aβ accumulation precedes plaque formation in β-amyloid precursor protein and presenilin-1 double-transgenic mice. Neurosci Lett 306:116–120. 10.1016/S0304-3940(01)01876-6 11403971 10.1016/s0304-3940(01)01876-6

[112] Wirths O, Multhaup G, Bayer TA (2004) A modified β-amyloid hypothesis: Intraneuronal accumulation of the β-amyloid peptide - the first step of a fatal cascade. J Neurochem 91:513–520. 10.1111/j.1471-4159.2004.02737.x 15485483 10.1111/j.1471-4159.2004.02737.x

[113] Wong TP, Marchese G, Casu MA, Ribeiro-Da-Silva A, Caludio Cuello A, De Koninck Y (2000) Loss of presynaptic and postsynaptic structures is accompanied by compensatory increase in action potential-dependent synaptic input to layer V neocortical pyramidal neurons in aged rats. J Neurosci 20:8596–8606. 10.1523/jneurosci.20-22-08596.2000 11069968 10.1523/JNEUROSCI.20-22-08596.2000 PMC6773180

[114] Yang W, Wang J, Guo J, Dove A, Qi X, Bennett DA, Xu W (2024) Association of Cognitive Reserve Indicator with Cognitive decline and structural brain differences in Middle and older age: findings from the UK Biobank. J Prev Alzheimer’s Disease 3:739–748. 10.14283/jpad.2024.54 10.14283/jpad.2024.54 PMC11061039 38706290

[115] Yankner BA, Duffy LK, Kirschner DA (1990) Neurotrophic and neurotoxic effects of amyloid β protein: reversal by Tachykinin Neuropeptides. Sci (1979) 250:279–282. 10.1126/science.2218531 10.1126/science.2218531 2218531

[116] Yin Y, Gao D, Wang Y, Wang Z-HH, Wang XCXX-C, Ye J, Wu D, Fang L, Pi G, Yang Y, Wang XCXX-C, Lu C, Ye K, Wang J-ZZ (2016) Tau accumulation induces synaptic impairment and memory deficit by calcineurin-mediated inactivation of nuclear CaMKIV/CREB signaling. Proceedings of the National Academy of Sciences 113:E3773–E3781. 10.1073/pnas.1604519113 10.1073/pnas.1604519113 PMC4932970 27298345

[117] Yoshiyama Y, Higuchi M, Zhang B, Huang SM, Iwata N, Saido TC, Maeda J, Suhara T, Trojanowski JQ, Lee VMY (2007) Synapse loss and Microglial Activation Precede tangles in a P301S Tauopathy Mouse Model. Neuron 53:337–351. 10.1016/j.neuron.2007.01.010 17270732 10.1016/j.neuron.2007.01.010

