# Supplementary figures and images for: Paradoxical attenuation of early amyloid-induced cognitive impairment and synaptic plasticity in an aged APP/Tau bigenic rat model
Source: Acta Neuropathol Commun. 2024 Dec 20;12:193. doi: 10.1186/s40478-024-01901-0 (PMC11662582; doi:10.1186/s40478-024-01901-0)

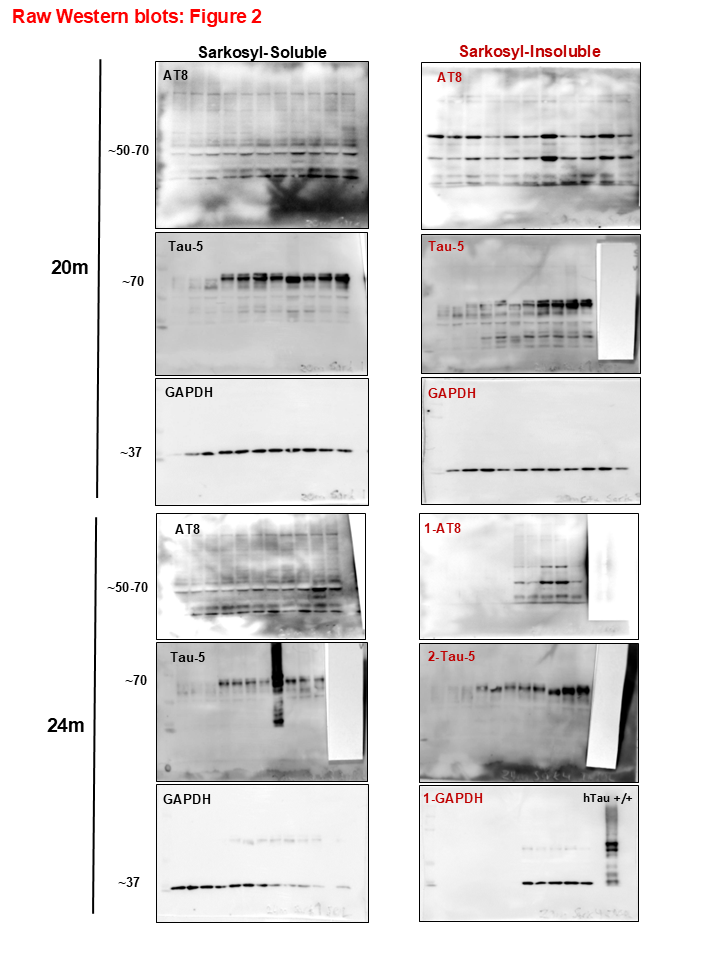

Supplement: Supplementary file 1 — Supplementary Material 1 [file 40478_2024_1901_MOESM1_ESM.tif]

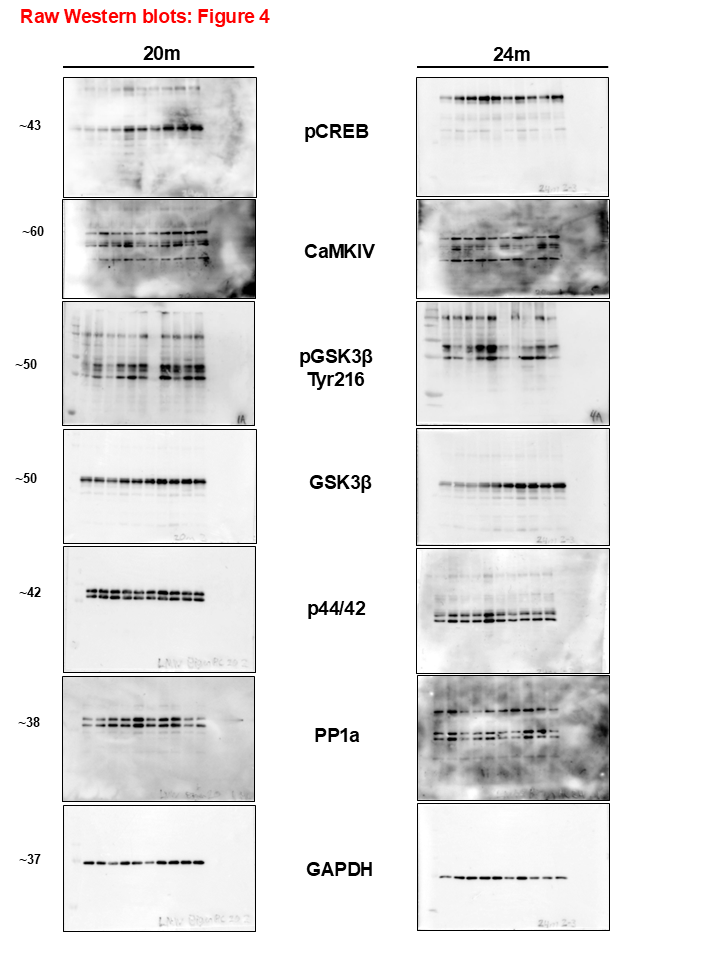

Supplement: Supplementary file 2 — Supplementary Material 2 [file 40478_2024_1901_MOESM2_ESM.tif]

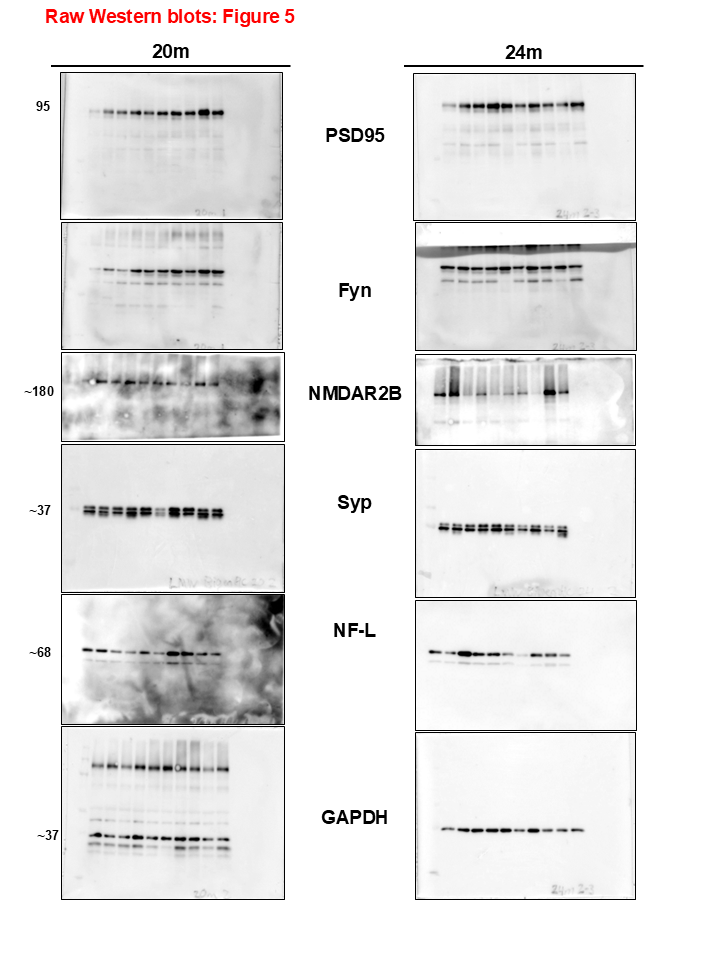

Supplement: Supplementary file 3 — Supplementary Material 3 [file 40478_2024_1901_MOESM3_ESM.tif]

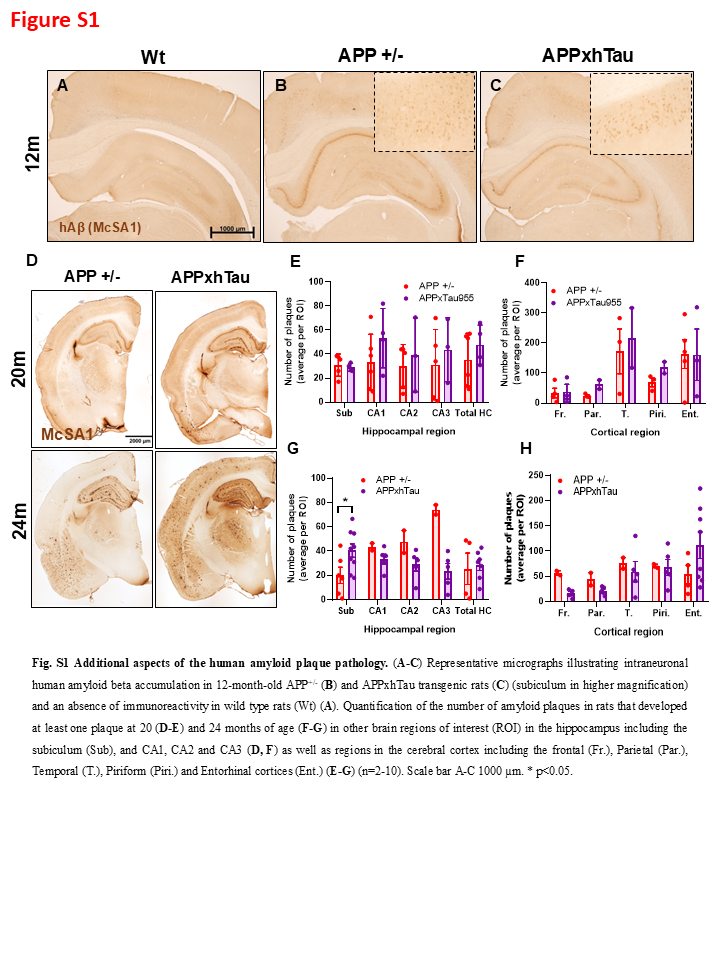

Supplement: Supplementary file 4 — Supplementary Material 4 [file 40478_2024_1901_MOESM4_ESM.tif]

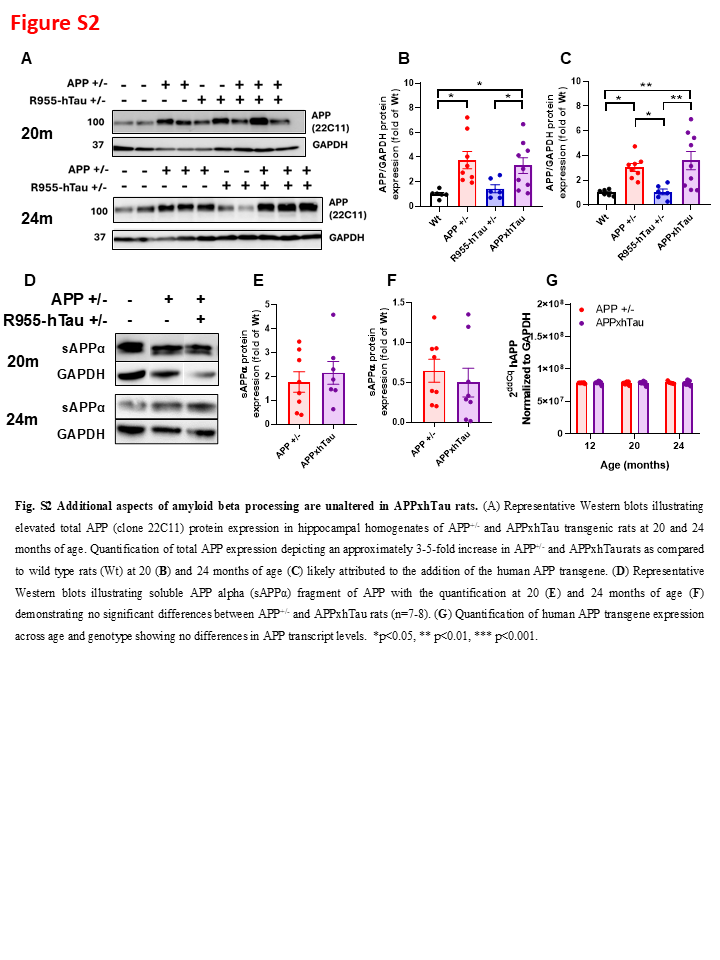

Supplement: Supplementary file 5 — Supplementary Material 5 [file 40478_2024_1901_MOESM5_ESM.tif]

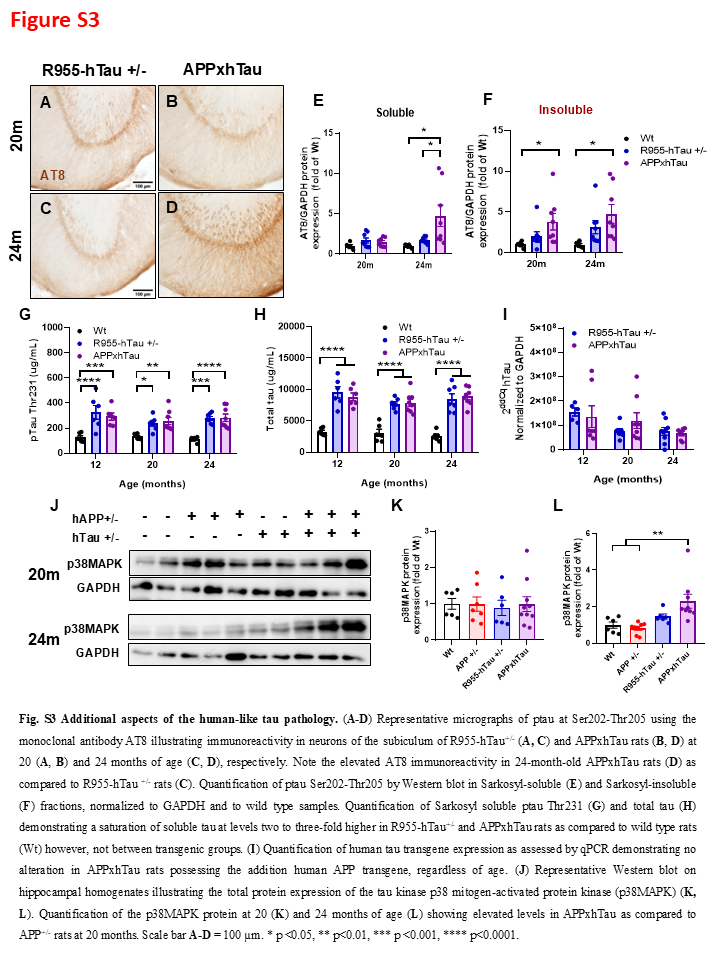

Supplement: Supplementary file 6 — Supplementary Material 6 [file 40478_2024_1901_MOESM6_ESM.tif]

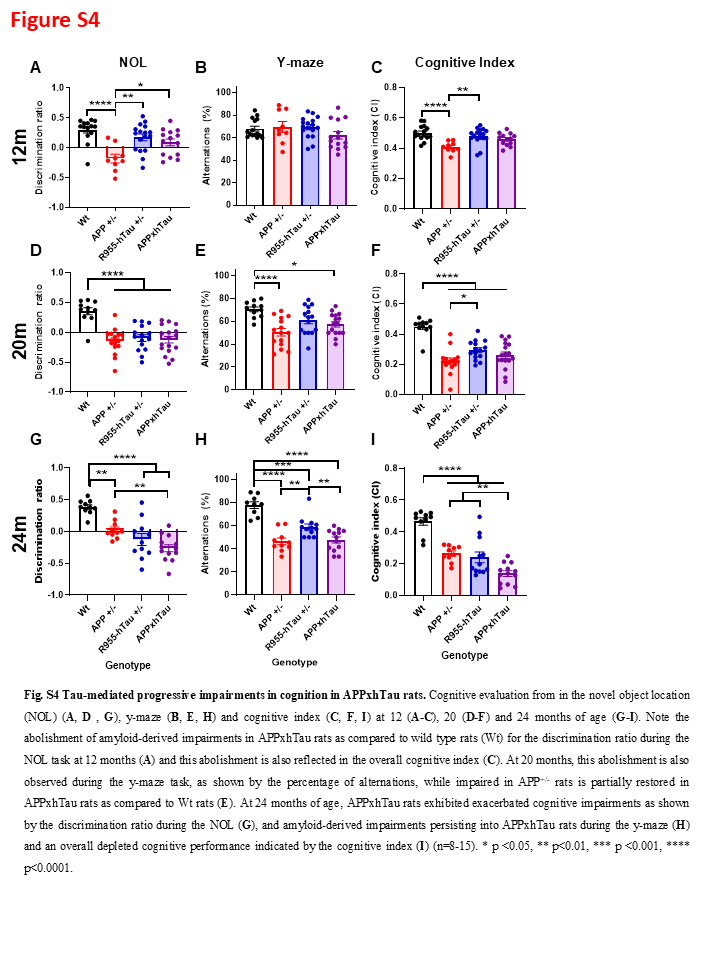

Supplement: Supplementary file 7 — Supplementary Material 7 [file 40478_2024_1901_MOESM7_ESM.tif]

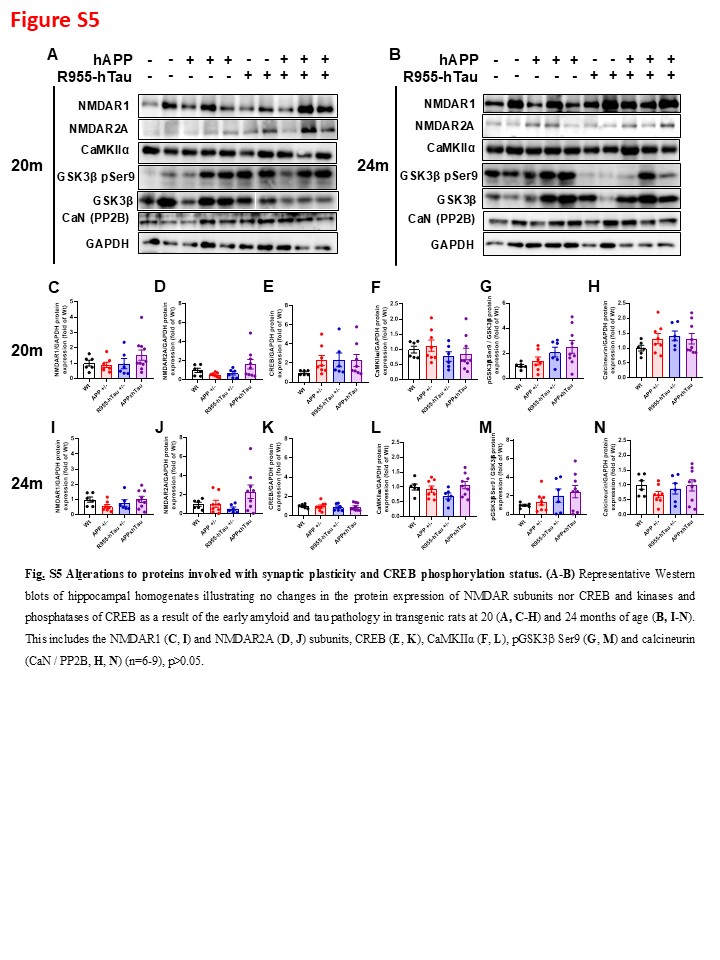

Supplement: Supplementary file 8 — Supplementary Material 8 [file 40478_2024_1901_MOESM8_ESM.tif]

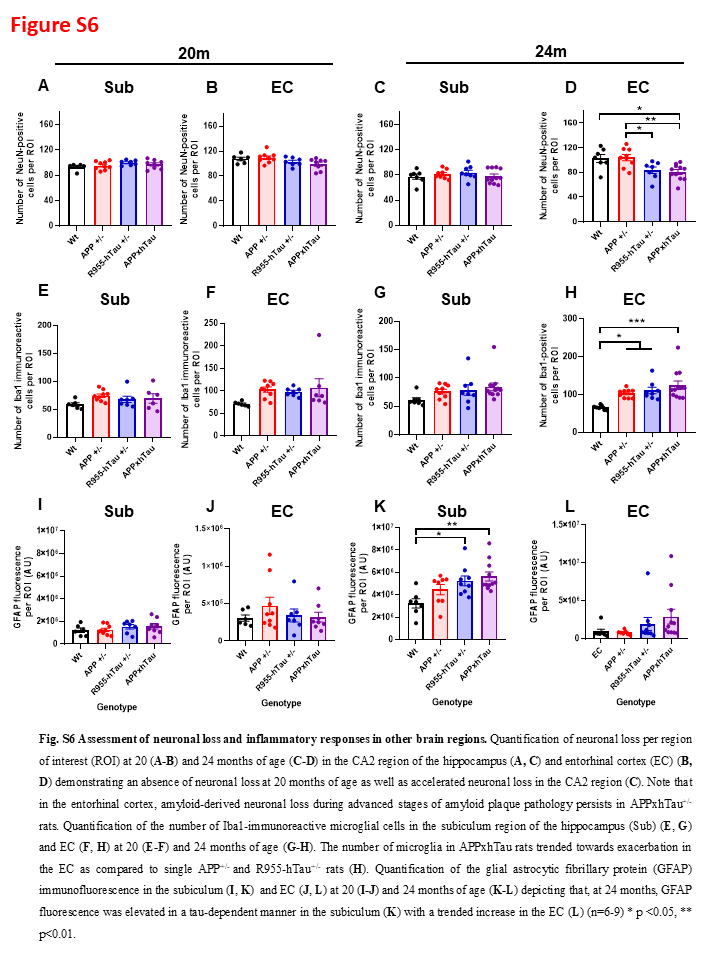

Supplement: Supplementary file 9 — Supplementary Material 9 [file 40478_2024_1901_MOESM9_ESM.tif]
