# Supplementary material for: Paradoxical attenuation of early amyloid-induced cognitive impairment and synaptic plasticity in an aged APP/Tau bigenic rat model
Source: Acta Neuropathol Commun. 2024 Dec 20;12:193. doi: 10.1186/s40478-024-01901-0 (PMC11662582; doi:10.1186/s40478-024-01901-0)
Supplement: Supplementary file 10 — Supplementary Material 10 [file 40478_2024_1901_MOESM10_ESM.docx]

**Fig. S1 Additional aspects of the human amyloid plaque pathology.** (**A-C**) Representative micrographs illustrating intraneuronal human amyloid beta accumulation in 12-month-old APP^+/−^ (**B**) and APPxhTau transgenic rats (**C**) (subiculum in higher magnification) and an absence of immunoreactivity in wild type rats (Wt) (**A**). Quantification of the number of amyloid plaques in rats that developed at least one plaque at 20 (**D-E**) and 24 months of age (**F-G**) in other brain regions of interest (ROI) in the hippocampus including the subiculum (Sub), and CA1, CA2 and CA3 (**D, F**) as well as regions in the cerebral cortex including the frontal (Fr.), Parietal (Par.), Temporal (T.), Piriform (Piri.) and Entorhinal cortices (Ent.) (**E-G**) (n = 2–10). Scale bar A-C 1000 µm. * p < 0.05.

**Fig. S2 Additional aspects of amyloid beta processing are unaltered in APPxhTau rats.** (A) Representative Western blots illustrating elevated total APP (clone 22C11) protein expression in hippocampal homogenates of APP^+/−^ and APPxhTau transgenic rats at 20 and 24 months of age. Quantification of total APP expression depicting an approximately 3-5-fold increase in APP^+/−^ and APPxhTau rats as compared to wild type rats (Wt) at 20 (**B**) and 24 months of age (**C**) likely attributed to the addition of the human APP transgene. (**D**) Representative Western blots illustrating soluble APP alpha (sAPPα) fragment of APP with the quantification at 20 (**E**) and 24 months of age (**F**) demonstrating no significant differences between APP^+/−^ and APPxhTau rats (n = 7–8). (**G**) Quantification of human APP transgene expression across age and genotype showing no differences in APP transcript levels. *p < 0.05, ** p < 0.01, *** p < 0.001.

**Fig. S3 Additional aspects of the human-like tau pathology.** (**A-D**) Representative micrographs of ptau at Ser202-Thr205 using the monoclonal antibody AT8 illustrating immunoreactivity in neurons of the subiculum of R955-hTau^+/−^ (**A, C**) and APPxhTau rats (**B**, **D**) at 20 (**A**, **B**) and 24 months of age (**C**, **D**), respectively. Note the elevated AT8 immunoreactivity in 24-month-old APPxhTau rats (**D**) as compared to R955-hTau ^+/−^ rats (**C**). Quantification of ptau Ser202-Thr205 by Western blot in Sarkosyl-soluble (**E**) and Sarkosyl-insoluble (**F**) fractions, normalized to GAPDH and to wild type samples. Quantification of Sarkosyl soluble ptau Thr231 (**G**) and total tau (**H**) demonstrating a saturation of soluble tau at levels two to three-fold higher in R955-hTau^+/−^ and APPxhTau rats as compared to wild type rats (Wt) however, not between transgenic groups. (**I**) Quantification of human tau transgene expression as assessed by qPCR demonstrating no alteration in APPxhTau rats possessing the addition human APP transgene, regardless of age. (**J**) Representative Western blot on hippocampal homogenates illustrating the total protein expression of the tau kinase p38 mitogen-activated protein kinase (p38MAPK) (**K, L**). Quantification of the p38MAPK protein at 20 (**K**) and 24 months of age (**L**) showing elevated levels in APPxhTau as compared to APP^+/−^ rats at 20 months. Scale bar **A-D** = 100 µm. * p < 0.05, ** p < 0.01, *** p < 0.001, **** p < 0.0001.

**Fig. S4 Tau-mediated progressive impairments in cognition in APPxhTau rats.** Cognitive evaluation from in the novel object location (NOL) (**A**, **D** , **G**), y-maze (**B**, **E**, **H**) and cognitive index (**C**, **F**, **I**) at 12 (**A-C**), 20 (**D-F**) and 24 months of age (**G-I**). Note the abolishment of amyloid-derived impairments in APPxhTau rats as compared to wild type rats (Wt) for the discrimination ratio during the NOL task at 12 months (**A**) and this abolishment is also reflected in the overall cognitive index (**C**). At 20 months, this abolishment is also observed during the y-maze task, as shown by the percentage of alternations, while impaired in APP^+/−^ rats is partially restored in APPxhTau rats as compared to Wt rats (**E**). At 24 months of age, APPxhTau rats exhibited exacerbated cognitive impairments as shown by the discrimination ratio during the NOL (**G**), and amyloid-derived impairments persisting into APPxhTau rats during the y-maze (**H**) and an overall depleted cognitive performance indicated by the cognitive index (**I**) (n = 8–15). * p < 0.05, ** p < 0.01, *** p < 0.001, **** p < 0.0001.

**Fig. S5 Alterations to proteins involved with synaptic plasticity and CREB phosphorylation status. (A-B)** Representative Western blots of hippocampal homogenates illustrating no changes in the protein expression of NMDAR subunits nor CREB and kinases and phosphatases of CREB as a result of the early amyloid and tau pathology in transgenic rats at 20 (**A, C-H**) and 24 months of age (**B, I-N**). This includes the NMDAR1 (**C**, **I**) and NMDAR2A (**D**, **J**) subunits, CREB (**E**, **K**), CaMKIIα (**F**, **L**), pGSK3β Ser9 (**G**, **M**) and calcineurin (CaN / PP2B, **H**, **N**) (n = 6–9), p > 0.05.

**Fig. S6 Assessment of neuronal loss and inflammatory responses in other brain regions.** Quantification of neuronal loss per region of interest (ROI) at 20 (**A-B**) and 24 months of age (**C-D**) in the CA2 region of the hippocampus (**A, C**) and entorhinal cortex (EC) (**B, D**) demonstrating an absence of neuronal loss at 20 months of age as well as accelerated neuronal loss in the CA2 region (**C**). Note that in the entorhinal cortex, amyloid-derived neuronal loss during advanced stages of amyloid plaque pathology persists in APPxhTau^+/−^ rats. Quantification of the number of Iba1-immunoreactive microglial cells in the subiculum region of the hippocampus (Sub) (**E**, **G**) and EC (**F**, **H**) at 20 (**E-F**) and 24 months of age (**G-H**). The number of microglia in APPxhTau rats trended towards exacerbation in the EC as compared to single APP^+/−^ and R955-hTau^+/−^ rats (**H**). Quantification of the glial astrocytic fibrillary protein (GFAP) immunofluorescence in the subiculum (**I**, **K**) and EC (**J**, **L**) at 20 (**I-J**) and 24 months of age (**K-L**) depicting that, at 24 months, GFAP fluorescence was elevated in a tau-dependent manner in the subiculum (**K**) with a trended increase in the EC (**L**) (n = 6–9) * p < 0.05, ** p < 0.01.

# **Supplemental Tables**

**Supplemental Table 1. Animal groups used throughout the study.**

| Age (months) | Wild type | McGill-APP +/- | McGill-R955-hTau +/- | APPxhTau | Total |
| --- | --- | --- | --- | --- | --- |
| 12 | 15 (9/6) | 10 (7/3) | 17 (12/5) | 14 (8/6) | 56 |
| 20 | 11 (4/7) | 15 (7/8) | 14 (7/7) | 20 (7/13) | 60 |
| 24 | 11 (4/7) | 11 (5/6) | 12 (4/8) | 14 (6/8) | 48 |
| Total  (M/F) | 37 (17/20) | 36 (19/17) | 43 (23/20) | 48 (21/27) | 164 |

**Supplemental Table 2. Frequency of plaque-positive animals.**

| Age (months) | Genotype | Hippocampus | Chi square | Cerebral Cortex | Chi square |
| --- | --- | --- | --- | --- | --- |
| **20** | **APP** +/-  **APPxhTau** | 6/15 (40 %)  4/13 (30 %) | p=0.6112 | 5/15 (33.3 %)  3/13 (23.1 %) | p=0.5491 |
| **24** | **APP** +/-  **APPxhTau** | 6/12 (50.0 %)  10/16 (62.5 %) | p=0.5083 | 4/12 (33.3 %)  9/16 (56.3 %) | p=0.2289 |

**Supplemental Table 3. List of antibodies used in the study.**

| Primary Antibody | Product ID | Company/Source | Epitope | Application | Concentration |
| --- | --- | --- | --- | --- | --- |
| **Amyloid beta** |  |  |  |  |  |
| hAPP (6e10) | 803001 | Biolegend | aa 3-8 | IF, WB | 1:1000 |
| APP (22C11) | MAB348 | EMD Millipore | aa 66-81 | WB | 1:1000 |
| McSA1 | MM-0015-P | Medimabs | hAβ aa 1-12 | IHC, IF | 1:1000 |
| Anti β-Amyloid | 8243 | Cell Signalling Tech | - | IF | 1:500 |
| sAPPα (2B3) | 11088 | IBL America | - | WB | 1:500 |
|  |  |  |  |  |  |
| **Tau, ptau** |  |  |  |  |  |
| HT7 | MN1000 | Thermofisher | aa 159-163, hTau | IHC | 1:1200 |
| AT8 | MN1020 | Thermofisher | pSer202-pThr205 | IHC, WB | 1:500, 1:1000 |
| MC1 | - | Peter Davies | aa 7-9, 312-322 | IHC | 1:500 |
| PHF1 | - | Peter Davies | pSer396/404 | IHC | 1:4000 |
| AT180 | MN1040 | Thermofisher | pThr231 | IHC | 1:500 |
| Tau-5 | AHB0042 | Thermofisher | - | WB | 1:1000 |
| **Cell Markers** |  |  |  |  |  |
| Iba1 (Microglia) | 019-19741 | Wako | - | IHC, IF | 1:500 |
| GFAP (Astrocyte) | NBP2-34354 | Novus | - | IF | 1:2000 |
| NeuN (Neuron) | Ab177487 | Abcam | - | IHC, IF | 1:4000 |
|  |  |  |  |  |  |
| **Synaptic** |  |  |  |  |  |
| NMDAR1 | G8913 | Sigma | - | WB | 1:1000 |
| NMDAR2A | 4205 | Cell Signalling Tech | - | WB | 1:1000 |
| NMDAR2B | 14544 | Cell Signalling Tech | - | WB | 1:1000 |
| PSD-95 | 3409 | Cell Signalling Tech | - | WB | 1:1000 |
| Fyn | Ab125016 | Abcam | - | WB | 1:1000 |
| Synaptophysin | 101-011 | Synaptic Systems | - | WB | 1:2000 |
|  |  |  |  |  |  |
|  |  |  |  |  |  |
| **Other** |  |  |  |  |  |
| GSK3B (27C10) | 9315 | Cell Signalling Tech | - | WB | 1:1000 |
| pGSK3B Ser9 | 9336 | Cell Signalling Tech | - | WB | 1:1000 |
| pGSK3B Tyr216 | 44-604G | Invitrogen | - | WB | 1:1000 |
| p38MAPK | 9212 | Cell Signalling Tech | - | WB | 1:1000 |
| p-p38MAPK (Thr180/Tyr182) | 9215 | Cell Signalling Tech | - | WB | 1:1000 |
| p44MAPK (Erk1/2) | 9102 | Cell Signalling Tech | - | WB | 1:1000 |
| Neurofilament L | ab223343 | Abcam | - | WB | 1:1500 |
| Creb | 9197 | Cell Signalling Tech |  | WB | 1:1000 |
| pCreb (Ser133) | 9196 | Cell Signalling Tech | - | WB | 1:1000 |
| CaMKIV | 4032 | Cell Signalling Tech | - | WB | 1:1000 |
| CaMKIIα | ab22609 | Abcam | - | WB | 1:1000 |
| PP1α | 2582 | Cell Signalling Tech |  | WB | 1:1000 |
| Calcineurin (CaN/PP2B) | 2614 | Cell Signalling Tech | - | WB | 1:1000 |
| CRTC1 | 2587 | Cell Signalling Tech | - | IF | 1:200 |
| GAPDH | MAB374 | Millipore | - | WB | 1:5000 |
|  |  |  |  |  |  |
| **Secondary Antibody** |  |  |  |  |  |
| Rabbit anti-Fc Mouse* | - | Cuello Lab | - | IHC | 1:100 |
| Goat anti-Rabbit-Biotinylated | BA-1000-1.5 | Vector Labs | - | IHC | 1:200 |
| Goat anti-Rabbit-HRP | 086-743-71 | Jackson Labs | - | WB | 1:5000 |
| Goat anti-Mouse-HRP | 115-035-003 | Jackson Labs | - | WB | 1:5000 |
| Goat anti-Rabbit AF-568 | A-11011 | Invitrogen | - | IF | 1:400 |
| Goat anti-Mouse AF-488 | A-11001 | Invitrogen | - | IF | 1:400 |

**IF** = Immunofluorescence; **IHC** = Immunohistochemistry; **WB** = Western blot
* Anti-Fc mouse refers to only the constant fragment (Fc) region of the mouse monoclonal antibody, reducing the possibility of nonspecific interactions from other components of anti-mouse secondary antibodies.
